# Total Synthesis and Structural Reassignment of the Antitubercular Natural Product Evybactin

**DOI:** 10.1002/chem.202403767

**Published:** 2024-11-14

**Authors:** Vladyslav Lysenko, Sangkeun Son, Monique E. Theriault, Cornelis J. Slingerland, Glenn Hauk, Laurence Cleenewerk, Alexander Speer, James M. Berger, Kim Lewis, Nathaniel I. Martin

**Affiliations:** ^1^ Biological Chemistry Group Institute of Biology Leiden University Leiden The Netherlands; ^2^ Antimicrobial Discovery Center Department of Biology Northeastern University Boston, MA USA; ^3^ Department of Biophysics and Biophysical Chemistry Johns Hopkins University School of Medicine Baltimore, MD USA; ^4^ Department of Molecular Microbiology A-LIFE AIMMS Vrije Universiteit Amsterdam Amsterdam The Netherlands; ^5^ Department of Medical Microbiology and Infection Prevention Amsterdam University Medical Centre VU Medical Center Amsterdam The Netherlands

**Keywords:** Evybactin, Antibiotic, Tuberculosis, Total synthesis, Structural assignment

## Abstract

The escalating threat posed by antibiotic resistance is a global concern and underscores the need for new antibiotics. In this context, the recent discovery of evybactin, a nonribosomal depsipeptide antibiotic that selectively and potently inhibits the growth of *M. tuberculosis*, is particularly noteworthy. Here, we present the first total synthesis of this natural product, along with a revision of its assigned structure. Our studies revealed a disparity between the structure originally proposed for evybactin and its actual configuration. Specifically, the 3‐methylhistidine residue present in the evybactin core macrocycle was found to be of the d‐configuration rather than the previously assigned l‐His(Me). Having addressed this, we further optimized our solid‐phase synthetic route to provide access to evybactin on a multi‐hundred‐milligram scale. Access to such quantities will allow for more comprehensive studies with this promising antibiotic.

## Graphical abstract

We here report the first total synthesis of the antitubercular natural product evybactin, together with the structural reassignment of the previously proposed structure. The optimized synthetic method developed provides access to evybactin on the multi‐hundred‐milligram scale and provides the opportunity for future structure‐activity relationship studies.

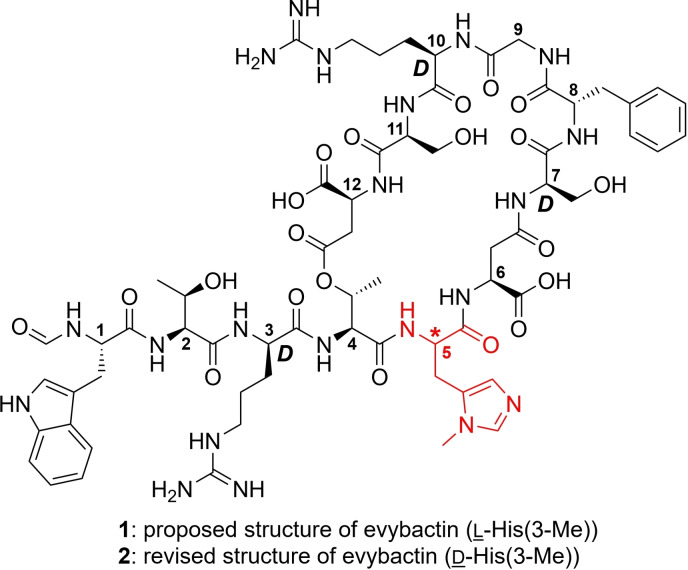

## Introduction

Globally, tuberculosis (TB) is responsible for millions of infections each year and, with the exception of the recent COVID‐19 pandemic period, has historically been the pathogen with the single highest attributable annual death toll. The standard treatment for TB infection typically involves daily administration of multi‐drug cocktails for many months, often resulting in poor compliance and the emergence of resistance.[^1^, ^2^] The threat of extensively drug‐resistant strains of TB underscores the need for innovative antibacterial therapies to counter these and other serious bacterial pathogens.[^3^, ^4^, ^5^, ^6^] In recent years, screening uncultured bacteria^[7]^ or targeting of microbiome symbionts such as *Photorhabdus* has led to the discovery of compounds with unique antibacterial mechanisms of action.^[8]^ Among recent demonstrations of this strategy is the Lewis group's discovery of evybactin ‐ a unique DNA gyrase inhibitor that selectively and potently inhibits the growth of TB.^[9]^

**Figure 1:**
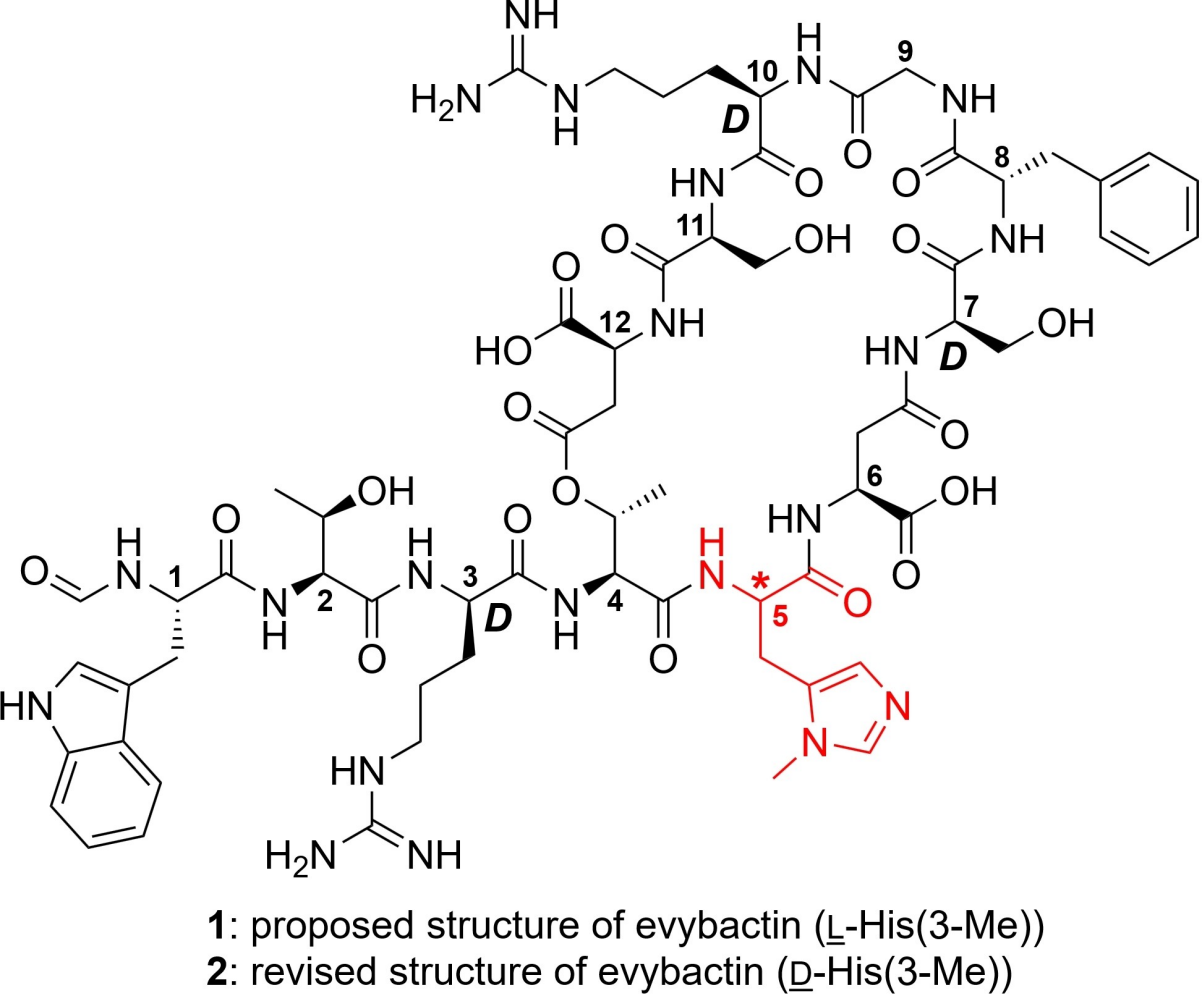
Originally proposed structure of evybactin (**1**) and revised structure (**2**) established via total synthesis.

Isolated from culture extracts of the nematode microbiome symbionts *Photorhabdus*, evybactin was found to be a nonribosomal depsipeptide containing 12 amino acids. Extensive NMR and MS/MS studies combined with analysis of the biosynthetic gene cluster responsible for the biosynthesis of evybactin led to a proposed structure (Figure 1).^[9]^

Evybactin was found to contain a unique macrocyclic structure formed between the side chain carboxyl group of the C‐terminal Asp^12^ residue and the side chain hydroxyl of Thr^4^. Also of note, within the macrocycle, it is the side chain carboxyl group of Asp^6^ that forms a peptide bond with neighboring d‐Ser^7^. In addition to these features, evybactin was also found to contain d‐Arg residues at positions 3 and 10, along with N‐terminal formylation at Trp^1^ and methylation at N3 in the imidazole side‐chain of His^5^.

Evybactin's unique narrow spectrum activity against TB was shown to be due to its capacity to gain entry by hijacking the BacA transporter, used by TB as a multi‐solute ABC‐type transporter for hydrophilic molecules^[10]^ such as vitamin B_12_.^[11]^ Upon cell entry, evybactin acts as a DNA gyrase poison, with structural studies subsequently showing that it binds to a recently discovered site that is also exploited by a novel class of synthetic thiophene antibiotics.[^9^, ^12^] Evybactin's specific entry mechanism and strong gyrase inhibition are responsible for its potent antibacterial activity against TB, with minimum inhibitory concentration (MIC) values as low as 0.25 μg/mL while exhibiting no measurable toxicity against mammalian cells.^[9]^

While evybactin's promising anti‐TB activity merits further exploration, its isolation from natural sources presents a barrier to the investigation and exploitation of its potential. Isolation of evybactin from fermentation of *P. noenieputensis* is labor‐intensive, requiring complex isolation and purification procedures and yields small quantities of material.^[9]^ Furthermore, access to analogs to enable structure‐activity relationship (SAR) studies is not readily achievable starting from the natural product. For these reasons, we were motivated to develop a synthetic route that would provide access to high‐purity evybactin in larger quantities and provide a means for generating structural analogs.

## Results and Discussion

Given our previous experience in the synthesis of macrocyclic peptide antibiotics,[^13^, ^14^, ^15^, ^16^, ^17^] we elected to pursue a microwave‐assisted solid phase peptide synthesis (SPPS) approach for the preparation of evybactin. To this end, we first explored a direct route wherein a protected linear 12‐mer peptide was synthesized starting from the C‐terminal Asp^12^ (based on the numbering in Figure 1) loaded via its side‐chain carboxyl group onto 2‐chlorotrityl chloride (2‐CTC) resin (see supplemental Scheme S1). In preparing the linear precursor peptide we used 10 % piperazine in *N*‐methyl‐2‐pyrrolidone (NMP)/ethanol (EtOH) for Fmoc deprotections and *N*,*N*,*N*′,*N*′‐tetramethyl‐O‐(1*H*‐benzotriazol‐1‐yl)uronium hexafluorophosphate (HBTU) and DIPEA for amino acid couplings. We also used Fmoc‐Thr‐OH (with the side‐chain hydroxyl unprotected) for the installation of Thr^4^, which did not interfere with any of the subsequent couplings. Following the final Fmoc deprotection, the N‐terminus of Trp^1^ was cleanly formylated on resin using a mixture of 4‐nitrophenylformate and DIPEA in DMF. ^[18]^ Given the limited stability of the formylation reagent, this step was performed manually at room temperature. The protected peptide was then cleaved from the resin using hexafluoroisopropanol (HFIP) to maintain all side‐chain protecting groups. Next, we explored a variety of conditions for closing the macrocycle via ester bond formation between the free side‐chain hydroxyl of Thr^4^ and the free side‐chain carboxyl of Asp^12^. Our initial attempt using dicyclohexylcarbodiimide/4‐dimethylaminopyridine (DIC/DMAP) failed to yield any of the cyclized product, however upon addition of 1,8‐diazabicyclo[5.4.0]undec‐7‐ene (DBU) we observed partial conversion to a species with the expected mass. However, following global deprotection using trifluoroacetic acid/triisopropylsilane/water (TFA/TIPS/H_2_O), the crude peptide contained many closely eluting impurities that precluded purification of the desired product. Switching to different cyclization conditions using O‐(7‐azabenzotriazol‐l‐yl)‐*N*,*N*,*N*′,*N*′‐tetramethyluronium hexafluorophosphate (HATU), and DBU also resulted in incomplete conversion and again yielded complex mixtures preventing purification.

**Scheme 1:**
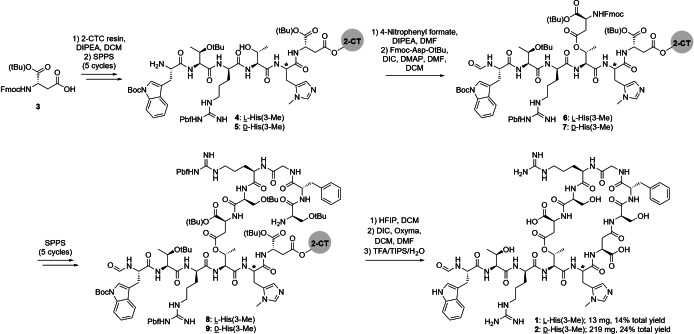
SPPS route developed for the synthesis of evybactin. Boc=tert‐Butyloxycarbonyl, DCM=dichloromethane, DIC=N,N‐diisopropylcarbodiimide, DIPEA=diisopropylethylamine, DMAP=4‐dimethylaminopyridine, DMF=dimethylformamide, Fmoc=fluorenylmethoxycarbonyl, HFIP=hexafluoroisopropanol, Oxyma=ethyl cyanohydroxyiminoacetate, Pbf=2,2,4,6,7‐pentamethyldihydrobenzofuran‐5‐sulfonyl, TFA=trifluoroacetic acid, TIPS=triisopropylsilane.

We next opted for a strategy wherein the ester linkage between Thr^4^ and Asp^12^ was formed on resin at an earlier stage of the synthesis, followed by closure of the macrocycle by subsequent amide formation between d‐Ser^7^ and Asp^6^ (Scheme 1). In doing so, we started by loading Fmoc‐Asp‐OtBu **3** (corresponding to Asp^6^) onto 2‐CTC resin via its side‐chain carboxyl group. Five rounds of SPPS resulted in the resin bound intermediate hexapeptide **4** terminating in Trp^1^. As in our previous route, we used Fmoc‐Thr‐OH (with the side‐chain hydroxyl unprotected) for the installation of Thr^4^, which did not affect the quality of any of the following couplings. On resin formylation of the N‐terminus of Trp^1^ was performed manually at room temperature, after which the hydroxyl side‐chain of Thr^4^ was esterified with Fmoc‐Asp‐OtBu (also under manual conditions) using DIC/DMAP activation in DMF/DCM, resulting in a reasonable 85 % conversion to intermediate **6** as assessed by LCMS analysis. Automated SPPS was again used for the installation of the remaining amino acids, yielding the resin bound intermediate **8** terminating at d‐Ser^7^. The peptide was then cleaved from the resin using HFIP treatment to maintain all side‐chain protection. The crude protected peptide was then directly treated with DIC/Oxyma (6 : 6) in DCM/DMF (6 : 1), which gratifying was found to result in the clean and complete formation of the desired macrocycle by amide bond formation between the amine of d‐Ser^7^ and the side‐chain carboxyl of Asp^6^. Following this, global deprotection with TFA/TIPS/H_2_O and subsequent purification using reverse‐phase high‐performance liquid chromatography (RP‐HPLC) resulted in a yield of 13 mg of compound **1** (14 % overall yield, corresponding to an average yield of 93 % per step).

**Figure 2:**
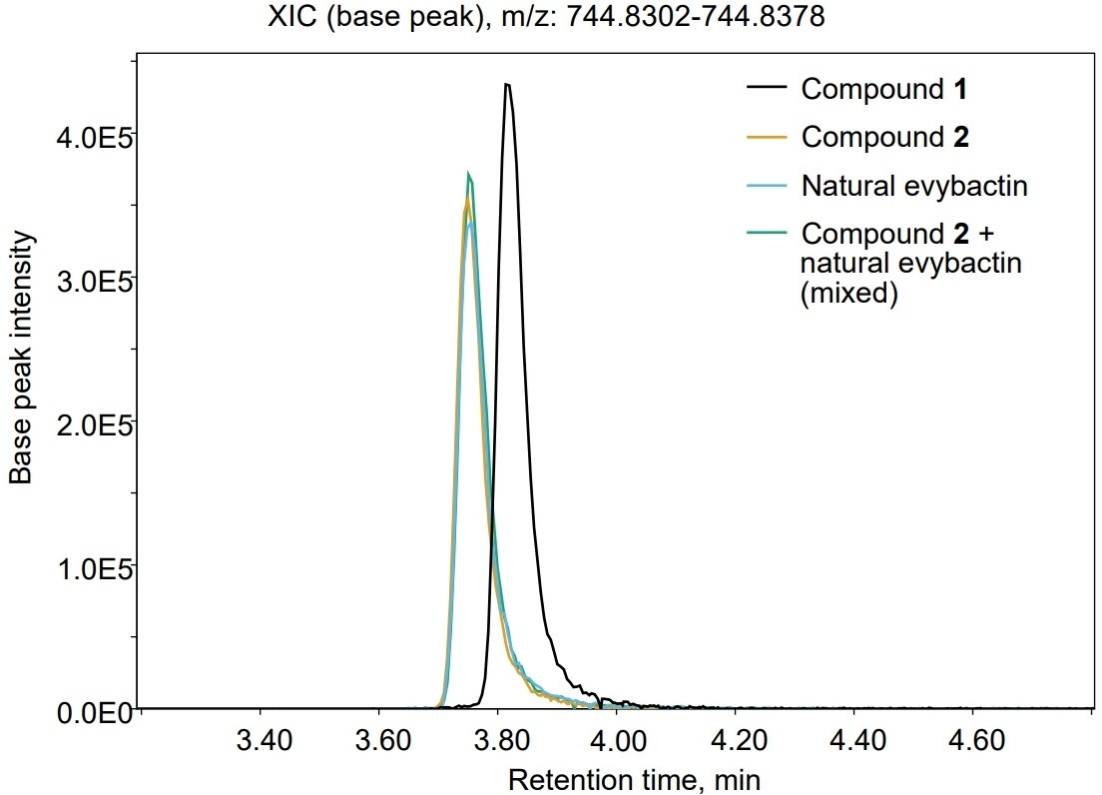
Extracted ion chromatograms of authentic evybactin (m/z=744.8344 for [M+2H]^2+^) isolated from fermentation of *P. noenieputensis* overlaid with traces obtained for synthetic compounds **1** and **2**. Compound **2** co‐elutes with natural product. Traces for authentic evybactin in blue, compound **1** in black, compound **2** in yellow, and the combination of authentic evybactin + compound **2** in green (detection range for doubly charged species m/z=744.8302–744.8378).

We next proceeded to characterize the antibacterial activity of compound **1** and, to our surprise, found it to be completely inactive against *M. tuberculosis* H37Rv mc^2^6020, *E. coli* ATCC 25922, and *E. coli* MG1655 while the natural product isolated from fermentation of *P. noenieputensis*, exhibited the same activity against these strains as previously reported (Table 1).^[9]^ Compound **1** was also found to be much less active against the hypersensitive *E. coli* WO153 strain with increased membrane permeability. Subsequent investigations revealed that **1** is not identical to the natural product based on LC–MS co‐injection experiments (Figure 2) and a comparison of the NMR data acquired (see supplemental Figures S1‐S2). These findings, and the lack of antibacterial activity observed for **1**, suggested that the structure originally assigned to evybactin might require revision. To this end, we first assessed the amino acid connectivity of evybactin by performing and comparing MS/MS analyses for the natural product and compound **1**. The results obtained from these studies confirmed the originally assigned connectivity, with that of natural evybactin matching that of compound **1** (see supplemental Table S1‐S2). These findings prompted us to further investigate the stereochemistry assigned for the different amino acids present in evybactin. To this end, we performed Marfey‘s analysis^[19]^ on both the natural product and **1**. Notably, the data thus obtained suggested that the stereochemistry of the His(3‐Me) residue at position 5 was d‐ rather than l‐ as originally assigned (see supplemental Table S3). This led to a reanalysis of the biosynthetic gene cluster of evybactin, which, upon closer inspection, was also found to support the presence of d‐His(3‐Me). Specifically, antiSMASH analysis^[20]^ of the 12 nonribosomal peptide synthetases (NRPS) modules encoding evybactin revealed a previously unassigned epimerase domain downstream of module 5, responsible for the incorporation of Me‐His (see supplemental Figure S4).

**Table 1:** Minimum inhibitory concentrations determined for evybactin isolated from fermentation of *P. noenieputensis* compared with synthetic compounds **1** and **2**.

MIC (μg/mL)			
	Evybactin (natural)	**1**	**2**
*M. tuberculosis* H37Rv mc^2^6020^[a]^	0.25	128	0.25
*E. coli* ATCC 25922^[b]^	8	>128	8
*E. coli* MG1655^[b]^	16	>128	16
*E. coli* WO153 (AB1157; *recJ asmB1 ΔtolC*::KanR) ^[b]^	0.0625	0.8	0.0625
*S. aureus* HG003^[b]^	128	>128	>128

[a] Difco Middlebrook 7H9 medium; [b] MHIIB medium.

Equipped with these insights, we next proceeded to synthesize compound **2** (Figure 1) according to the route developed for **1** (Scheme 1) but with the notable incorporation of d‐His(3‐Me) at position 5. Interestingly, while Fmoc‐l‐His(3‐Me)‐OH is widely available, the corresponding d‐enantiomer could not be readily sourced from commercial suppliers. The published methods for synthesizing Fmoc‐d‐His(3‐Me)‐OH were found to be complicated and low‐yielding.[^21^, ^22^] We, therefore, elected to develop a new protocol for the preparation of this key building block with the aim of pursuing a more convenient, chromatography‐free route (see supplemental Scheme S3). Starting from readily available Fmoc‐d‐His(Trt)‐OH, we first protected the carboxyl group as the Trt ester by treatment with TrtCl and DIPEA. Following a simple extractive work up, treatment with MeI in DMF was found to cleanly alkylate position 3 of the imidazole ring. Subsequent removal of both Trt groups by treatment with TFA/TIPS/H_2_O, followed by precipitation using methyl t‐butyl ether (MTBE)/petroleum ether (PE), provided Fmoc‐d‐His(3‐Me)‐OH in 92 % yield over 3 steps. This procedure was found to be reproducible at a gram scale, providing the building block in purity suitable for direct use in SPPS.

The synthesis of **2** was carried out as for **1**, with some minor adjustments that were found to improve the overall yield. Specifically, for the transformation of resin bound intermediate **5** to **7**, we found that reducing the number of formylation cycles from 3 to 2 led to suppression of an unwanted side product presumably formed due to formylation of the side‐chain hydroxyl of Thr^4^. We also found that for the subsequent esterification of the Thr^4^ side chain with Fmoc‐Asp‐OtBu, a lower amount of DMAP (0.2 eq) improved the conversion, as higher concentrations of DMAP resulted in premature Fmoc deprotection at Asp^12^ leading to unwanted side products. As for the cyclization step, we found that the original extractive work‐up used resulted in a significant loss of material and negatively impacted the yield. To avoid this, we reduced the number of DIC/Oxyma equivalents to 3 and decreased the concentration of DMF in DCM to 0.5 %, which allowed for direct evaporation of the solvent after the cyclization was complete (as established by LCMS analysis). The material thus obtained was then deprotected by direct treatment with TFA/TIPS/H_2_O followed by precipitation of the product from MTBE. This optimized protocol both improved the purity of the crude product (which greatly facilitated final purification by RP‐HPLC) and resulted in an increased overall yield for compound **2** of 24 % (corresponding to an average yield of 94.8 % per step). This method was found to be reproducible and performed well at 0.5 mmol scale, providing access to quantities of **2** in excess of 200 mg.

With compound **2** in hand, we next proceeded to assess its antibacterial activity against the same panel of strains previously used for natural evybactin and compound **1**. We were delighted to find that the activity profile of compound **2** completely matches that of authentic evybactin (Table 1). In addition, the NMR spectra (see supplemental Figures S1‐S3) and MS data obtained for compound **2** match those of the natural product along with co‐injection experiments (Figure 2), further confirming that the structure of evybactin is indeed that of compound **2**.

**Figure 3:**
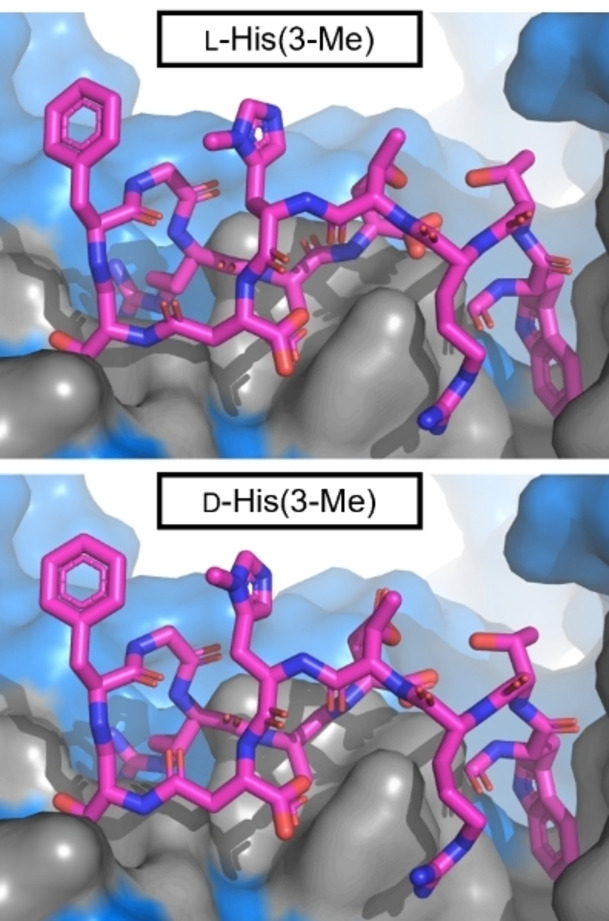
Crystal structure of evybactin bound to DNA gyrase from *M. tuberculosis*, modeled with compounds **1** and **2** containing l‐ vs. d‐methyl histidine stereoisomer, respectively (PDBID 7UGW). Gyrase is depicted in light blue surface representation, with the contact surface (residues within 4 Å) for evybactin colored in grey. Evybactin structures are illustrated as sticks with carbons in magenta.

Having confirmed that the structure of evybactin contains d‐His(3‐Me) at position 5, we also revisited the previously reported crystal structure of the evybactin‐DNA gyrase complex (Figure 3).^[9]^ Somewhat surprisingly, the change in stereochemistry at d‐His(3‐Me)^5^ was not found to have a significant impact on the modeled conformation of evybactin when bound to gyrase. As indicated by the revised model, it appears that the side chain of d‐His(3‐Me)^5^ does not make key contacts with the enzyme, which in turn raises the question as to why there is such a pronounced difference in the antibacterial activity for the position 5 epimers **1** and **2**. A possible explanation may be that the BacA transporter, responsible for the active transport of evybactin into the bacterial cell, may have a strong preference for the natural product. In support of this explanation is the finding that unnatural l‐His(3‐Me)^5^ containing **1** does exhibit antibacterial activity against *E. coli* WO153 (MIC =0.8 μg/mL), a strain with a hyperpermeable outer membrane along with knockout of the TolC porin to which MDR pumps dock (Table 1). While authentic evybactin is clearly more active against this strain (MIC=0.0625 μg/mL), these findings suggest that if able to gain entry to the cell, the unnatural analog may also be able to engage with the gyrase in a manner similar to the natural product.

## Conclusions

In conclusion, we here report the first total synthesis of the potent antitubercular natural product evybactin. Our synthetic studies led to the reassignment of the stereochemical configuration of the His(3‐Me) residue at position 5 to the d‐configuration in contrast to the l‐configuration in the originally proposed structure. Synthetic evybactin containing d‐His(3‐Me) at position 5 was found to have the same antibacterial activity profile as the natural product, along with matching analytical data. Notably, our synthetic route provides a convenient method for the preparation of evybactin at a scale that will enable studies aimed at establishing its therapeutic potential. Furthermore, the convenient SPPS based route reported here can also provide access to evybactin analogs for SAR studies. The evaluation of evybactin in relevant *in vivo* models of disease, along with the design and synthesis of novel evybactin analogs, are ongoing in our laboratories and will be reported in due course.

## Supporting Information Summary

Experimental details, compound characterization data, copies of NMR spectra, HPLC traces. The authors have also cited additional references within the Supporting Information.[^9^, ^19^, ^23^, ^24^, ^25^, ^26^]

## Conflict of Interests

The authors declare no conflict of interest.

1

## Supporting information

As a service to our authors and readers, this journal provides supporting information supplied by the authors. Such materials are peer reviewed and may be re‐organized for online delivery, but are not copy‐edited or typeset. Technical support issues arising from supporting information (other than missing files) should be addressed to the authors.

Supporting Information

## Data Availability

The data that support the findings of this study are available in the supplementary material of this article.
