# Supplementary material for: Total Synthesis and Structural Reassignment of the Antitubercular Natural Product Evybactin
Source: Chemistry. 2024 Nov 14;31(1):e202403767. doi: 10.1002/chem.202403767 (PMC11711294; doi:10.1002/chem.202403767)
Supplement: Supplementary file 1 — Supporting Information [file CHEM-31-e202403767-s001.pdf]

Chemistry–A European Journal

Supporting Information

Total Synthesis and Structural Reassignment of the Antitubercular Natural Product Evybactin

Vladyslav Lysenko, Sangkeun Son, Monique E. Theriault, Cornelis J. Slingerland, Glenn Hauk, Laurence Cleenewerk, Alexander Speer, James M. Berger, Kim Lewis, and Nathaniel I. Martin*

Supporting Information

Table of contents

	Page
**General information**	3-4
**Detailed experimental procedures**	5-9
**LC-MS/MS analysis**	10-11
**Marfey`s analysis**	12
**NMR comparison of authentic evybactin and synthetic compounds 1 and 2**	13-15
**Analysis of the biosynthetic gene cluster**	16
**NMR Spectra**	17-24
**HPLC analysis of peptides**	25
**References**	26

General information

Reagents

All reagents employed were of American Chemical Society (ACS) grade or higher and were used without further purification unless otherwise stated. Fmoc-D-Arg(Pbf)-OH, Fmoc-Asp-OtBu, Fmoc-D-His(Trt)-OH, Fmoc-D-Ser(tBu)-OH, Fmoc-Thr-OH, and 4-Nitrophenyl formate were purchased from Combi-Blocks. All other Fmoc-amino acids were purchased from P3 BioSystems. 2-Chloro trityl chloride (2-CT) resin and Fmoc-His(3-Me)-OH were purchased from Iris Biotech. *N*,*N*-Diisopropylcarbodiimide (DIC), ethyl cyanohydroxyiminoacetate (Oxyma), hexafluoroisopropanol (HFIP), *N*,*N*,*N′*,*N′*-Tetramethyl-O-(1*H*-benzotriazol-1-yl)uronium hexafluorophosphate (HBTU) and triisopropylsilane (TIPS) were purchased from Manchester Organics. 4-Dimethylaminopyridine (DMAP) and iodomethane (MeI) were purchased from Sigma Aldrich. Piperazine was purchased from Thermo Scientific. Trityl chloride (TrtCl) was purchased from Fluorochem. Diisopropylethylamine (DIPEA) and dimethyl sulfoxide (DMSO) were purchased from Carl Roth. Dichloromethane (DCM), diethyl ether (Et_2O), ethanol (EtOH), and petroleum ether (PE) were purchased from VWR Chemicals. Acetonitrile (ACN), dimethylformamide (DMF), methyl tertiary-butyl ether (MTBE), *N*-methyl-2-pyrrolidone (NMP), and trifluoroacetic acid (TFA) were purchased from Biosolve.

HRMS

High-resolution mass spectra (HRMS) analyses were performed on a Shimadzu Nexera X2 UHPLC system with a Waters Acquity HSS C18 column (2.1 × 100 mm, 1.8 μm) at 30 °C and equipped with a diode array detector. The following solvent system, at a flow rate of 0.5 mL/min, was used: solvent A, 0.1 % formic acid in water; solvent B, 0.1 % formic acid in acetonitrile. Gradient elution was as follows: 95:5 (A/B) for 1 min, 95:5 to 15:85 (A/B) over 10 min, 15:85 to 0:100 (A/B) over 1 min, 0:100 (A/B) for 4 min, then reversion back to 95:5 (A/B) for 3 min. This system was connected to a Shimadzu 9030 QTOF mass spectrometer (ESI ionisation) calibrated internally with Agilent's API-TOF reference mass solution kit (5.0 mM purine, 100.0 mM ammonium trifluoroacetate and 2.5 mM hexakis(1*H*,1*H*,3*H*-tetrafluoropropoxy)phosphazine) diluted to achieve a mass count of 10000.

Analytical HPLC

HPLC analyses were performed on a Shimadzu Prominence-i LC-2030 system with a Dr. Maisch ReproSil Gold 120 C18 column (4.6 × 250 mm, 5 μm) at 30 °C and equipped with a UV detector monitoring at 214 and 254 nm. The following solvent system, at a flow rate of 1 mL/min, was used: solvent A, 0.1 % TFA in water/acetonitrile 95/5; solvent B, 0.1 % TFA in water/acetonitrile 5/95. Gradient elution was as follows: 100:0 (A/B) for 3 min, 100:0 to 0:100 (A/B) over 47 min, 0:100 (A/B) for 4 min, then reversion back to 100:0 (A/B) over 1 min, 100:0 (A/B) for 5 min.

NMR

^{1}H and ^{13}C NMR spectra were recorded on Bruker AV 400 MHz (at 400.2 (^{1}H) and 100.6 (^{13}C) MHz), AV 600 MHz (at 600.1 (^{1}H) and 150.9 (^{13}C) MHz). The temperatures of the NMR experiments were 298K or 320K. Chemical shifts are reported in ppm (δ) and were calibrated using residual deuterated solvent as an internal reference. (δ ^{1}H NMR: DMSO 2.50, δ ^{13}C NMR: DMSO 39.52). The NMR data are processed as follows: chemical shift, multiplicity (s = singlet, d = doublet, dd = double doublet, t = triplet, dt = double triplet, q = quartet, tt = triple triplet, m = multiplet), integration, coupling constants (*J*, reported in Hz) and a number of nuclei. NMR spectra were analyzed and processed using MestreNova version 14.2.0.

MIC of synthetic evybactins

M. tuberculosis H37Rv mc26020 (ΔlysA ΔpanCD) expressing mCherry was grown in complete 7H9 OADC media to mid-log. Serial 2-fold dilutions of evybactins in water were made the day of assay, accounting for a final 10% of total assay volume. Mtb was added to a final OD600 of 0.01 in fresh 7H9 OADC media. Plates were incubated for 7 days at 37 °C prior to analyses via fluorescent plate reader of mCherry fluorescence. MICs were assigned

as the concentration where there was at least >90% inhibition relative to negative (water) control values. For MICs against E. coli and S. aureus, overnight cultures of E. coli MG1655, E. coli 25922, E. coli WO153, and S. aureus HG003 in Mueller-Hinton II broth (MHIIB) were diluted 1:100 and cultured at 37 °C with aeration at 220 rpm. After 2 h of incubation, the exponential phase cultures were further diluted to an OD600 of 0.001. Then, 98 µl of this diluted culture was added to 96-well plates containing 2 µl of serial 2-fold dilutions of evybactins in water. The activity was evaluated after 20 h of incubation at 37 °C. MICs were defined as the lowest concentration at which no visible cell growth was observed.

X-ray crystallography

Refinement restraints for evybactin containing D-methyl histidine were generated using the ELBOW PHENIX software package. D-methyl histidine was then manually re-fit into electron density using the previous L-methyl histidine evybactin structure as a guide.[9] Refinement was conducted using PHENIX refine, restricted to atoms within evybactin.

Detailed procedures for synthesis

Resin loading

2-Chlorotrityl chloride resin (2-CTC) (4 g, 1.51 mmol/g) was loaded by coupling via the free sidechain carboxyl group of Fmoc-Asp-OtBu (4.97 g, 12.08 mmol, 2 eq.) with DIPEA (5.26 mL, 30.2 mmol, 5 eq.) in 70 mL of CH_2Cl_2. After 3h at room temperature, an extra amount of DIPEA (5.26 mL, 30.2 mmol, 5 eq.) and 24 mol of MeOH were added, and the reaction was left shaking for another 15 minutes. The resin was then filtered, washed with DMF, EtOH, DCM, Et_2O, and dried overnight under a stream of N_2. The resin loading was then determined to be 0.558 mmol/g.

Automated solid-phase peptide synthesis

A CEM Liberty Blue automated peptide synthesizer with microwave irradiation was used to perform solid-phase peptide synthesis (SPPS). The next system was used for couplings: 0.05 mmol scale – 5eq. HBTU (0.25M in DMF), 10eq. DIPEA (0.5M in DMF), 5eq. of amino acid (0.2M in DMF); 0.5 mmol scale – 4eq. HBTU (0.25M in DMF), 8eq. DIPEA (0.5M in DMF), 4eq. of amino acid (0.2M in DMF). Fmoc group removal was performed using Piperazine:EtOH:NMP (1:1:9, m/v/v). A detailed overview of the automated protocols can be found below.

Resin swelling

The resin was swollen in 10 mL of DMF for 300 s prior to the first coupling.

Protocol 1: Standard Coupling protocol

Step	Function	Duration/Temperature
**1**	Deprotection N-terminus	50 s at 25°C then 250 s at 50°C
**2**	Wash (DMF)	RT
**3**	Wash (DMF)	RT
**4**	Wash (DMF)	RT
**5**	Coupling amino acid	150 s at 25°C then 750 s at 50°C

Protocol 2: 3-Me Histidine coupling protocol

Step	Function	Duration/Temperature
**1**	Deprotection N-terminus	50 s at 25°C then 250 s at 50°C
**2**	Wash (DMF)	RT
**3**	Wash (DMF)	RT
**4**	Wash (DMF)	RT
**5**	Coupling amino acid	1800 s at 25°C then 300 s at 50°C

Protocol 3: Arg coupling protocol

Step	Function	Duration/Temperature
**1**	Deprotection N-terminus	50 s at 25°C then 250 s at 50°C
**2**	Wash (DMF)	RT
**3**	Wash (DMF)	RT
**4**	Wash (DMF)	RT
**5**	Coupling amino acid	2400 s at 25°C
**6**	Coupling amino acid	1800 s at 25°C then 300 s at 50°C

Protocol 4: L-Ser coupling protocol

Step	Function	Duration/Temperature
**1**	Deprotection N-terminus	400 s at 25°C
**2**	Deprotection N-terminus	1200 s at 25°C
**3**	Wash (DMF)	RT
**4**	Wash (DMF)	RT
**5**	Wash (DMF)	RT
**6**	Coupling amino acid	3600 s at 25°C

Protocol 5: Final deprotection N-terminus

Step	Function	Duration/Temperature
**1**	Deprotection N-terminus	50 s at 25°C then 250 s at 50°C
**2**	Wash (DMF)	RT
**3**	Wash (DMF)	RT
**4**	Wash (DMF)	RT

Attempted synthesis of compound 1 via linear SPPS strategy

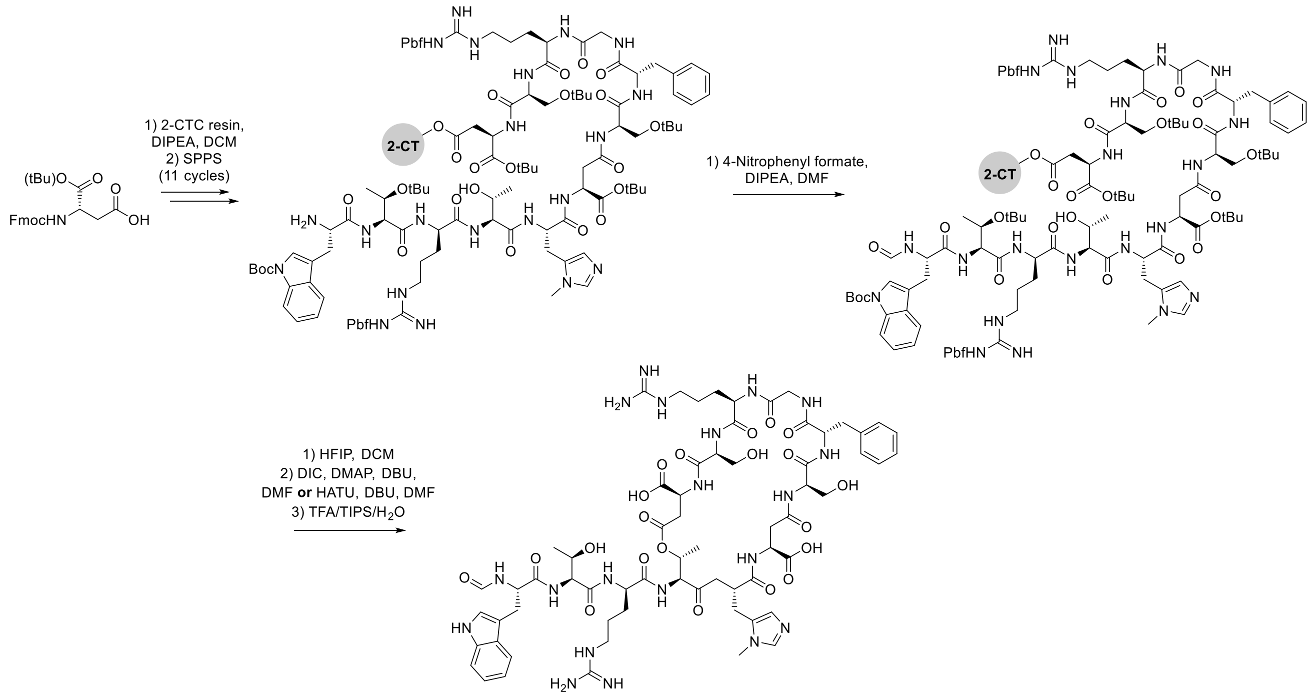

Scheme S1. Initially attempted synthesis of compound **1** that resulted in incomplete conversion and yielded complex mixtures preventing proper purification

Synthesis of the compounds 1 and 2 – proposed and revised structures of evybactin

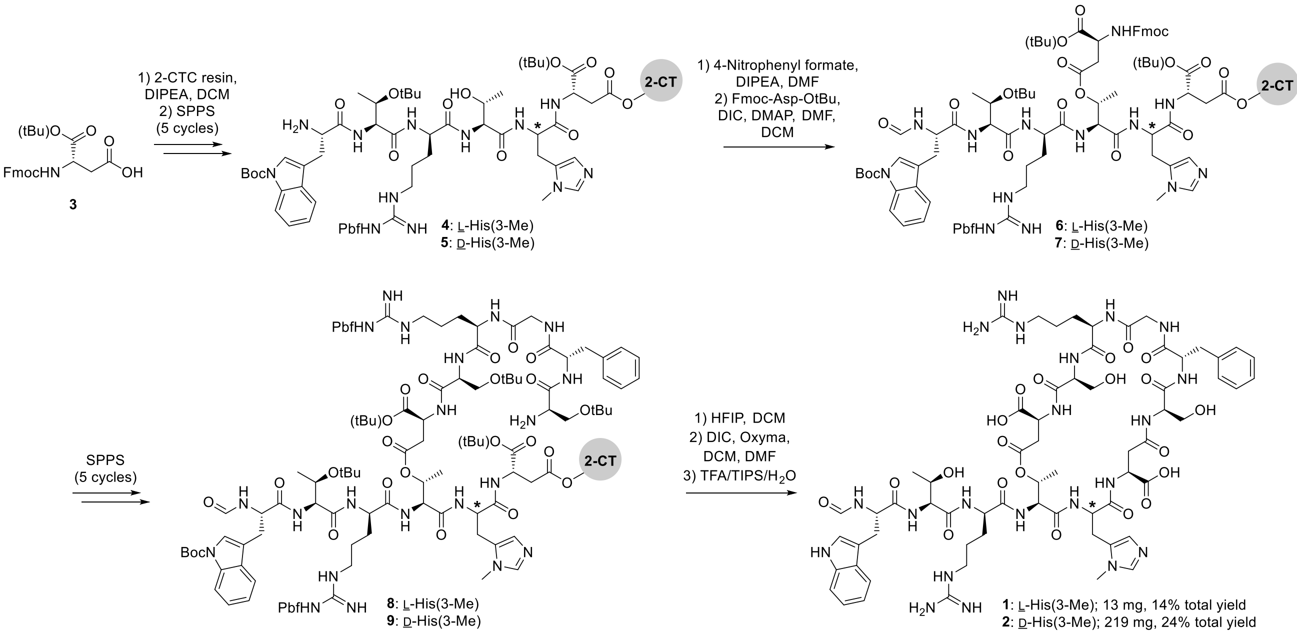

Scheme S2. SPPS route developed for the synthesis of compounds **1** and **2**

Procedure for the synthesis of the compound 1 – proposed structure of evybactin

2-CT resin loaded with Fmoc-Asp-OtBu was transferred into a CEM Liberty Blue µwave peptide synthesizer at a 0.05 mmol scale. The amino acids were coupled in the following order: 1) Fmoc-His(3-Me)-OH using protocol 2; 2) Fmoc-Thr-OH using protocol 1; 3) Fmoc-D-Arg(Pbf)-OH using protocol 3; 4) Fmoc-Thr-OH using protocol 1; 5) Fmoc-Trp(Boc)-OH using protocol 1 followed by a final deprotection using protocol 5. The resin was then transferred to a manual reactor for the SPPS connected to the nitrogen flow to perform formylation. After the resin was swollen in DMF (5 mL) for 1 min, 4-nitrophenyl formate (42 mg, 0.25 mmol) and DIPEA (88 mkl, 0.5 mmol) were added. After bubbling with N_2 for 30 min, the resin was filtered, washed with DMF (2 × 5 mL), and the formylation procedure was repeated 2 more times. After that, the resin was washed with DMF (2 × 5 mL), DCM (2 × 5 mL) and dried using N_2 flow. Fmoc-Asp-OtBu (206 mg, 0.5 mmol), was weighted out in the round bottom flask and diluted with 4 mL of dry DCM:DMF mixture (1:1), followed by the addition of DIC (79 mkl, 0.5 mmol). The mixture was left shaking for 1 h under Argon atmosphere. After that, DMAP (2.44 mg, 0.02 mmol) was added to the flask, followed by the addition of the dried resin after formylation. The reaction was left shaking overnight at room temperature under Argon atmosphere. After the reaction was finished the resin was filtered, washed with DMF (2 × 5 mL), DCM (2 × 5 mL) and transferred to the CEM Liberty Blue µwave peptide synthesizer to perform the next coupling in the following order: 1) Fmoc-Ser(tBu)-OH using protocol 4; 2) Fmoc-D-Arg(Pbf)-OH using protocol 3; 3) Fmoc-Gly-OH using protocol 1; 4) Fmoc-Phe-OH using protocol 1; 5) Fmoc-D-Ser(tBu)-OH using protocol 1 followed by final deprotection using protocol 5. The peptide was then detached from the resin with 6 mL of the 20% hexafluoroisopropanol (HFIP) in DCM. The solvents were removed by rotary evaporation, yielding the protected peptide, which was used directly in the next step.

Dry DMF (10 mL) was poured into the round-bottom flask containing a stirring bar, protected peptide (0.05 mmol), and Oxyma (42.6 mg, 0.3 mmol), followed by the addition of dry DCM (60 mL). The DIC (47 mkl, 0.3 mmol) was added to the resulting mixture, and the reaction was left stirring overnight. The next day the solvents were evaporated under reduced pressure, and the residue was diluted with water (20 mL) and extracted with EtOAc (2 × 30 mL). The combined organic layers were washed with 1M $NaHCO_3$ (25 mL), water (25 mL), brine (20 mL), dried under Na_2SO_4, and concentrated using rotary evaporation. Final sidechain deprotection was carried out by treating the obtained powder with 3 mL of the TFA/TIPS/H_2O (95:2.5:2.5) mixture for 2.5 h. The

reaction mixture was precipitated in MTBE/Petroleum ether (1:1) and centrifuged (4500 rpm, 5 min). The pellet was then resuspended in MTBE/Petroleum ether (1:1) and centrifuged again (4500 rpm, 5 min). Finally, the pellet containing the crude peptide was dissolved in H_2O/ACN (95:5) and lyophilized overnight.

Peptide was purified using a BESTA-Technik system with a Dr. Maisch Reprosil Gold 120 C18 column (25 × 250 mm, 10 µm) and equipped with an ECOM Flash UV detector monitoring at 214 nm and 254 nm. The following solvent system, at a flow rate of 12 mL/min, was used: solvent A, 0.1 % TFA in water/acetonitrile 95/5; solvent B, 0.1 % TFA in water/acetonitrile 5/95. Gradient elution was as follows: 100:0 (A/B) for 3 min, 100:0 to 50:50 (A/B) over 48 min, 50:50 to 0:100 (A/B) over 0.5 min, 0:100 (A/B) for 4 min, then reversion back to 100:0 (A/B) over 1 min, 100:0 (A/B) for 4 min. The fractions contained the product were combined and lyophilized to obtain compound **1** as a white fluffy powder (13 mg, 14% yield, 3×TFA salt) with >95% purity as determined by HPLC.

HRMS (ESI) m/z calcd for $C_{64}H_{89}N_{21}O_{21}$+$2H^+$: 744.8344 ($[M+2H]^{2+}/2$); found: 744.8344.

Procedure for the synthesis of the compound 2 – revised structure of evybactin

2-CT resin loaded with Fmoc-Asp-OtBu was transferred into a CEM Liberty Blue µwave peptide synthesizer at a 0.5 mmol scale. The amino acids were coupled in the following order: 1) Fmoc-D-His(3-Me)-OH using protocol 2; 2) Fmoc-Thr-OH using protocol 1; 3) Fmoc-D-Arg(Pbf)-OH using protocol 3; 4) Fmoc-Thr-OH using protocol 1; 5) Fmoc-Trp(Boc)-OH using protocol 1 followed by a final deprotection using protocol 5. The resin was then transferred to a manual reactor for the SPPS connected to the nitrogen flow to perform formylation. After the resin was swollen in DMF (20 mL) for 1 min, 4-nitrophenyl formate (420 mg, 2.5 mmol) and DIPEA (880 mkl, 5 mmol) were added. After bubbling with N_2 for 20 min, the resin was filtered, washed with DMF (2 × 20 mL), and the formylation procedure was repeated 1 more time. After that, the resin was washed with DMF (2 × 20 mL), DCM (2 × 20 mL) and dried using N_2 flow. Fmoc-Asp-OtBu (2.06 g, 5 mmol) was weighted out in the round bottom flask and diluted with 30 mL of dry DCM:DMF mixture (1:1), followed by the addition of DIC (790 mkl, 5 mmol). The mixture was left shaking for 20 min under Argon atmosphere. After that, DMAP (12.2 mg, 0.1 mmol) was added to the flask, followed by the addition of the dried resin after formylation. The reaction was left shaking overnight at room temperature under Argon atmosphere. After the reaction was finished the resin was filtered, washed with DMF (2 × 20 mL), DCM (2 × 20 mL) and transferred to the CEM Liberty Blue µwave peptide synthesizer to perform the next coupling in the following order: 1) Fmoc-Ser(tBu)-OH using protocol 4; 2) Fmoc-D-Arg(Pbf)-OH using protocol 3; 3) Fmoc-Gly-OH using protocol 1; 4) Fmoc-Phe-OH using protocol 1; 5) Fmoc-D-Ser(tBu)-OH using protocol 1 followed by final deprotection using protocol 5. The peptide was then detached from the resin with 25 mL of the 20% hexafluoroisopropanol (HFIP) in DCM. The solvents were removed by rotary evaporation, yielding the protected peptide, which was used directly in the next step.

Dry DMF (2.5 mL) was poured into the round-bottom flask containing a stirring bar, protected peptide (0.5 mmol), and Oxyma (214 mg, 1.5 mmol), followed by the addition of dry DCM (500 mL). The DIC (235 mkl, 1.5 mmol) was added to the resulting mixture, and the reaction was left stirring overnight under an Argon atmosphere. The next day, the solvents were evaporated under reduced pressure, and the residue was treated with 20 mL of the TFA/TIPS/H_2O (95:2.5:2.5) mixture for 2.5 h. The reaction mixture was precipitated in MTBE/Petroleum ether (1:1) and centrifuged (4500 rpm, 5 min). The pellet was then resuspended in MTBE/Petroleum ether (1:1) and centrifuged again (4500 rpm, 5 min). Finally, the pellet containing the crude peptide was dried with N_2 flow and then redissolved in water and purified using a BUCHI Pure C-815 Flash system with a BUCHI EcoFlex C18 50µm spherical 80g column and equipped with UV detector monitoring at 214 nm and 254 nm. The following solvent system, at a flow rate of 60 mL/min, was used: solvent A, 0.1 % TFA in water/acetonitrile 95/5; solvent B, 0.1 % TFA in water/acetonitrile 5/95. Gradient elution was as follows: 100:0 (A/B) for 3 min, 100:0 to 50:50 (A/B) over 40 min, 50:50 to 0:100 (A/B) over 1 min, 0:100 (A/B) for 4 min, then reversion back to 100:0 (A/B) over 1.5 min, 100:0 (A/B) for 7 min. The fractions contained the product were combined and lyophilized to obtain compound **2** as a white fluffy powder (219 mg, 24% yield, 3×TFA salt) with >95% purity as determined by HPLC.

HRMS (ESI) m/z calcd for $C_{64}H_{89}N_{21}O_{21}$+$2H^+$: 744.8344 ($[M+2H]^{2+}/2$); found: 744.8350.

Procedure for the synthesis of the Fmoc-D-His(3-Me)-OH (13)

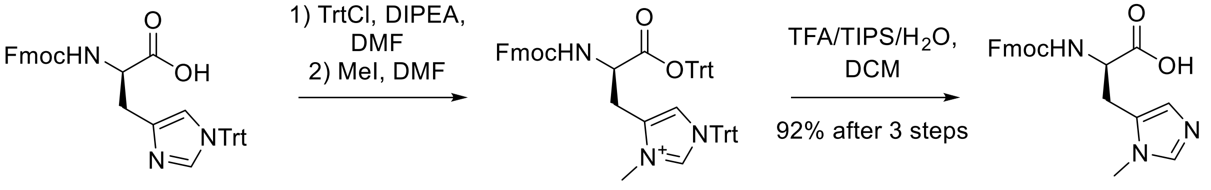

10 **11** **12**

Scheme S3: Route used for the synthesis of compound **12** (Fmoc-D-His(3-Me)-OH)

Fmoc-D-His(Trt)-OH (10 g, 16.1 mmol) was weighed out in the 100 mL round-bottom flask. The compound was dissolved with 50 mL of DMF, and then DIPEA (8.44 mL, 48.4 mmol) and TrtCl (9.9 g, 35.5 mmol) were added at RT. The mixture was left stirring for 3h, and after that, MeI (5.02 mL, 80.7 mmol) was added. The reaction was left stirring overnight. The next day, the mixture was poured into 300 mL of water, and the formed precipitate was filtered. The precipitate was then dissolved in DCM (200 mL) and brine (50 mL) and extracted. The organic layer was collected, dried under Na_2SO_4, and concentrated at reduced pressure to afford compound **11,** which was used directly in the next step without further purification.

The material from the previous step was dissolved in 100 mL of DCM, and then 100 mL of cleavage cocktail (TFA/TIPS/H2O = 95:2.5:2.5) was added. The reaction was left stirring for 3h. After that, the volume was reduced to 20 mL via evaporation, and the resulting mixture was poured into 300 mL of cold MTBE/PE (1:1) mixture. The formed precipitate was filtered, washed with MTBE (3 × 100 mL), and dried to obtain TFA salt of Fmoc-D-His(3-Me)-OH (**12**) (7.5 g, 92% overall yield) as a brown powder.

^{1}H NMR (400 MHz, DMSO-d_6, 298K): δ 14.49 (s, 1H), 13.34 (s, 1H), 9.01 (s, 1H), 7.89 (d, J = 7.6 Hz, 2H), 7.86 (d, J = 8.4 Hz, 1H), 7.69 – 7.62 (m, 2H), 7.41 (tt, J = 7.5, 1.7 Hz, 2H), 7.38 (d, J = 1.5 Hz, 1H), 7.32 (tt, J = 7.5, 1.4 Hz, 2H), 4.31 – 4.38 (m, 1H), 4.31 (d, J = 1.3 Hz, 1H), 4.29 (s, 1H), 4.20 (t, J = 6.9 Hz, 1H), 3.78 (s, 3H), 3.19 (dd, J = 15.9, 4.6 Hz, 1H), 3.02 (dd, J = 15.8, 10.1 Hz, 1H).

^{13}C{1H} NMR (101 MHz, DMSO-d_6, 298K) δ 172.2, 156.0, 143.8, 143.7, 140.8, 135.6, 131.2, 127.7, 127.1, 125.2, 120.2, 117.7, 65.7, 52.3, 46.6, 33.1, 24.8.

HRMS (ESI) m/z calcd for $C_{22}H_{21}N_3O_4$+H^+: 392.1605 $[M+H]^+$; found: 392.1617.

LC-MS/MS

For LC-MS analyses, compounds were dissolved at a concentration of 15 ug/mL in either H_2O/ACN (1:1) or 0.01M NaOH (followed by 3h incubation), and 1 µL was injected into Shimadzu Nexera X2 UHPLC system coupled to a Shimadzu 9030 QTOF mass spectrometer, and data acquisition was performed as previously described.[23] All the samples were analyzed in positive polarity, using data-dependent acquisition mode. In this regard, full scan MS spectra (m/z 100–1700, scan rate 10 Hz, ID enabled) were followed by two data-dependent MS/MS spectra (m/z 100–1700, scan rate 10 Hz, ID disabled) for the two most intense ions per scan. The ions were fragmented using collision-induced dissociation (CID) with fixed collision energy (CE 20 eV) and excluded for 1 s before being re-selected for fragmentation. The parameters used for the ESI source were: interface voltage 4 kV, interface temperature 300 °C, nebulizing gas flow 3 L/min, and drying gas flow 10 L/min. MZMine 3.2.8 [24] was used to analyze the obtained data.

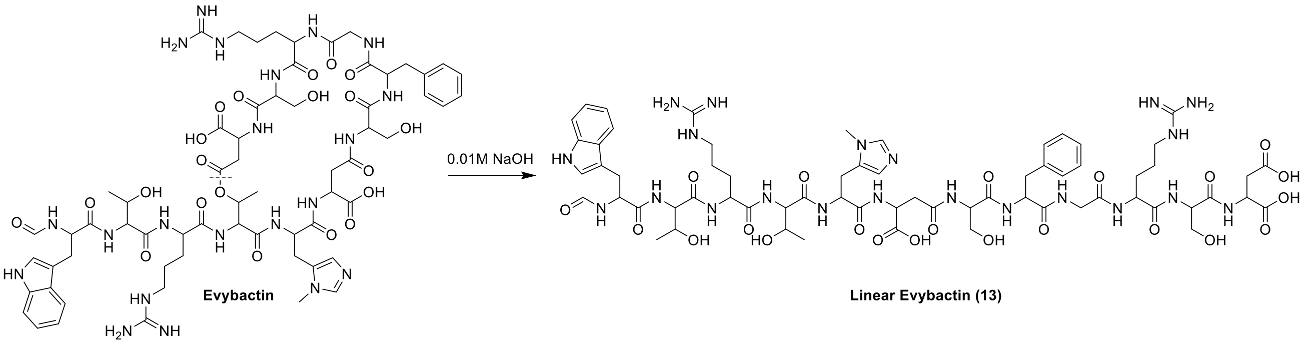

Scheme S4. Hydrolysis reaction of evybactin in prepared HRMS sample 0.01M solution of NaOH

Table S1. Comparing the synthetic compound **1** to an authentic evybactin sequence before and after the hydrolysis with NaOH by the combination of nearest-neighbor relationships (di-, tripeptide fragments) obtained. CO = Formyl group; H* = His(3-Me).

Dipeptides													Calc.	Found			
														**1**	**1** (NaOH)	Authentic evybactin	Authentic evybactin (NaOH)
CO	W												215.0815	215.8013	215.0816	215.0817	215.0816
	W	T											288.1343	288.1333	288.1355	288.1333	288.1339
		T	R										258.1561	258.1536	258.1574	258.1562	258.1562
			R	T									258.1561	258.1536	258.1574	258.1562	258.1562
				T	H*								253.1295	253.1303	253.1283	253.1299	253.1301
					H*	D							267.1088	267.1088	267.1087	267.1090	267.1090
						D	S						203.0662	203.0661	203.0657	203.0668	203.0667
							S	F					235.1077	235.1078	235.1076	235.1091	235.1085
								F	G				205.0972	205.0999	205.0982	205.0975	205.0955
									G	R			214.1299	214.1285	214.1315	214.1291	214.1278
										R	S		244.1404	244.1416	244.1421	244.1405	244.1418
											S	D	203.0662	203.0661	203.0657	203.0668	203.0667

Table S2. Comparing the synthetic compound **1** to an authentic evybactin sequence before and after the hydrolysis with NaOH by the combination of nearest-neighbor relationships (di-, tripeptide fragments) obtained. CO = Formyl group; H* = His(3-Me).

Dipeptides													Calc.	Found			
														**1**	**1** (NaOH)	Authentic evybactin	Authentic evybactin (NaOH)
CO	W	T											316.1292	316.1298	316.1290	316.1307	316.1293
	W	T	R										444.2354	444.2349	444.2332	444.2336	444.2352
		T	R	T									359.2037	NF	NF	NF	NF
			R	T	H*								409.2306	409.2327	409.2325	409.2321	409.2303
				T	H*	D							368.1565	368.1599	368.1570	368.1562	368.1570
					H*	D	S						354.1408	354.1432	354.1381	354.1396	354.1441
						D	S	F					350.1347	NF	NF	NF	NF
							S	F	G				292.1292	292.1292	292.1290	292.1297	292.1292
								F	G	R			361.1983	361.1976	361.1979	361.1982	361.1976
									G	R	D		301.1619	301.1621	301.1652	301.1629	301.1635
										R	S	D	359.1674	359.1681	359.1686	359.1671	359.1689

NF – not found

Marfey`s analysis

The stereochemistries of chiral centers present at α carbons were assigned by applying derivatization methods coupled with chromatographic analysis. The advanced Marfey's method using L-FDAA (1-fluoro-2-4-dinitrophenyl-5-L-alanine amide) established the absolute configurations of amino acids.[19]

The general method for Marfey`s analysis (as described):[25]

A sample of peptide (30 µg) in 6M HCl (100 µL) was heated to 100 °C in a sealed vial for 8−12 h, after which the hydrolysate was concentrated to dryness at 40 °C under a stream of dry N_2. The hydrolysate was then treated with 1 M $NaHCO_3$ (20 µL) and L-FDAA (1% solution in acetone, 40 µL) at 40 °C for 1 h, after which the reaction was neutralized with 1 M HCl (20 µL) and filtered (0.45 µm PTFE). An aliquot of the analyte was diluted 50 times with H_2O/ACN (1:1) and injected (2 µL) into an HRMS instrument following the standard protocol of the analysis (see general methods). The analyte amino acid content was assessed by comparison to authentic standards.

Table S3. Retention times (t_R, min) of the FDAA derivatives for authentic evybactin and standard amino acids.

	$[M+H]^+$	t_R, min			
		L-AA (standard)	D-AA (standard)	Authentic evybactin	Stereochemical assignment
Trp	457.1466	6.37	6.63	NF	**-**
Thr	372.1150	4.8	5.18	4.8	**L**
Arg	427.1684	4.27	4.23	4.23	**D**
His(3-Me)	422.1419	4.05	3.96	3.96	**D**
Asp	386.0943	4.81	4.91	4.81	**L**
Ser	358.0994	4.66	4.72	4.66/4.72	**L and D**
Phe	418.1357	6.51	6.9	6.51	**L**
Gly	328.0888	5.08	5.08	5.08	**NR**

NF – not found

NR – not relevant

NMR comparison of authentic evybactin and synthetic compounds 1 and 2

Conversion to the formic acid salt

The synthetic peptides **1** and **2** were converted from the TFA salt into the formic acid (FA) salt using the method below:

7 mg of the TFA salt of the previously purified peptide and 10 mg of the Na_2CO_3 were weighed out in the vial and diluted with 4 mL of water and 0.5 mL of the ACN. The mixture was purified using a BESTA-Technik system with a Dr. Maisch Reprosil Gold 120 C18 column (25 × 250 mm, 10 µm) and equipped with an ECOM Flash UV detector monitoring at 214 nm and 254 nm. The following solvent system, at a flow rate of 12 mL/min, was used: solvent A, 0.1 % FA in water/acetonitrile 95/5; solvent B, 0.1 % FA in water/acetonitrile 5/95. Gradient elution was as follows: 100:0 (A/B) for 3 min, 100:0 to 50:50 (A/B) over 48 min, 50:50 to 0:100 (A/B) over 0.5 min, 0:100 (A/B) for 4 min, then reversion back to 100:0 (A/B) over 1 min, 100:0 (A/B) for 4 min. The fractions contained in the product were combined and lyophilized to obtain compounds **1** or **2** as a white fluffy powder in quantitative yield with >95% purity as determined by HPLC.

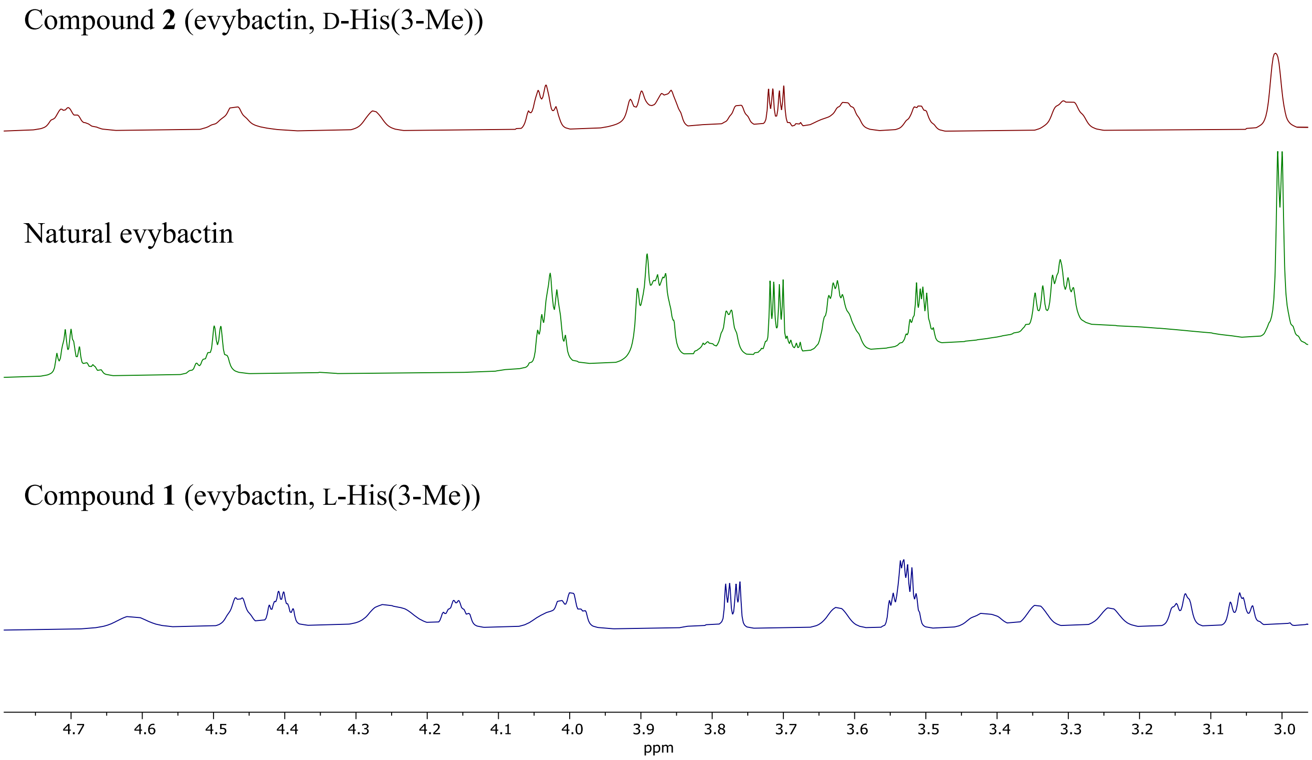

Figure S1. Alpha proton region (4.8 – 3.0 ppm) of the previously published ^{1}H-NMR (700 MHz, DMSO-d_6, 320K) spectrum of natural evybactin (as formic acid salt) isolated after fermentation of the producing organism overlaid with ^{1}H-NMR (600 MHz, DMSO-d_6, 320K) spectra of synthetic compounds **1** and **2** (as formic acid salts). The small difference between the NMR of compound **2** and that of natural evybactin can be explained by variations in the compound concentration and the content of water in the sample.

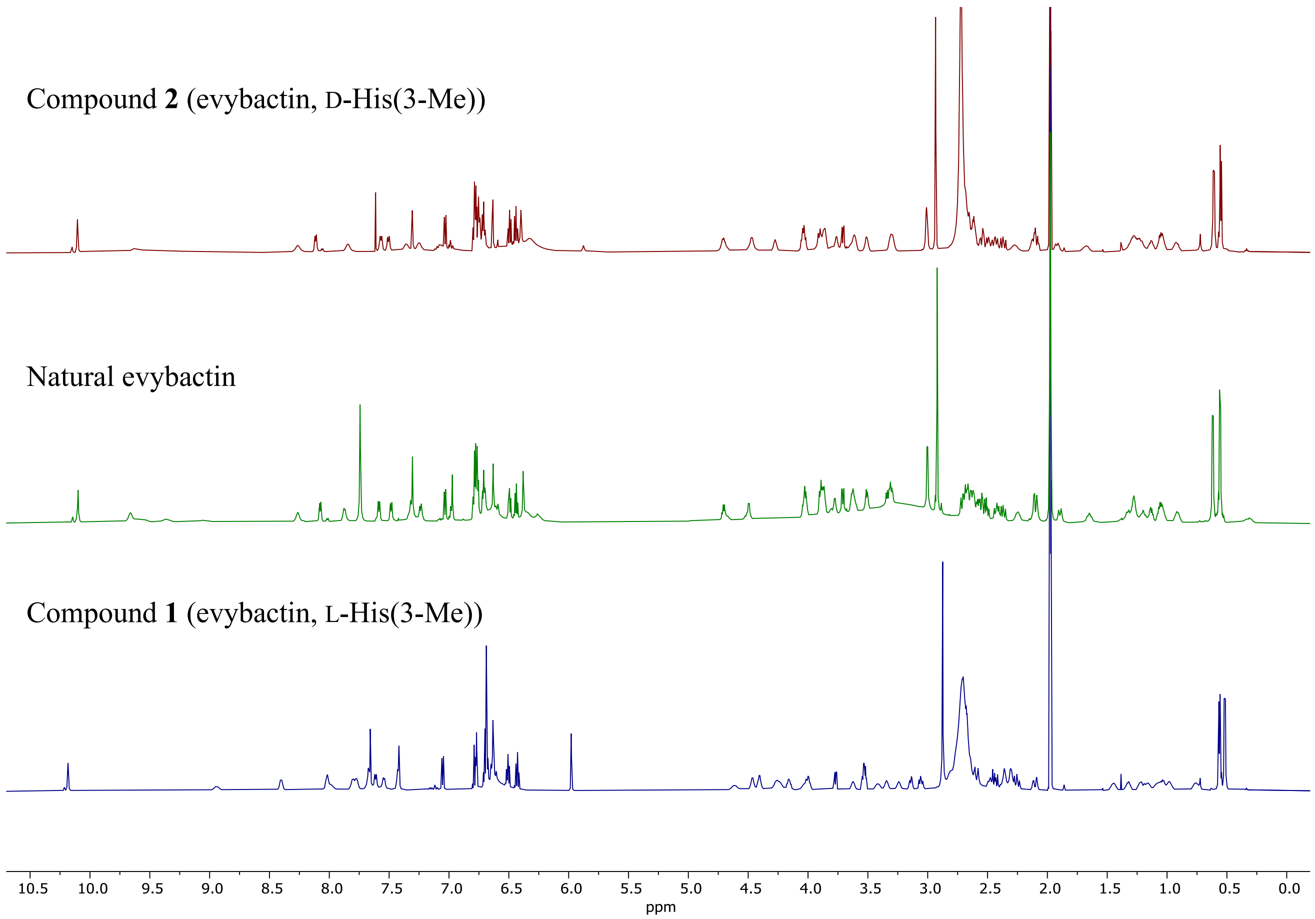

Figure S2. Full comparison (10.5 – 0 ppm) of the previously published ^{1}H-NMR (700 MHz, DMSO-d_6, 320K) spectrum of natural evybactin (as formic acid salt) isolated after fermentation of the producing organism overlaid with ^{1}H-NMR (600 MHz, DMSO-d_6, 320K) spectra of synthetic compounds **1** and **2** (as formic acid salts). The small difference between the NMR of compound **2** and that of natural evybactin can be explained by variations in the compound concentration and the content of water in the sample.

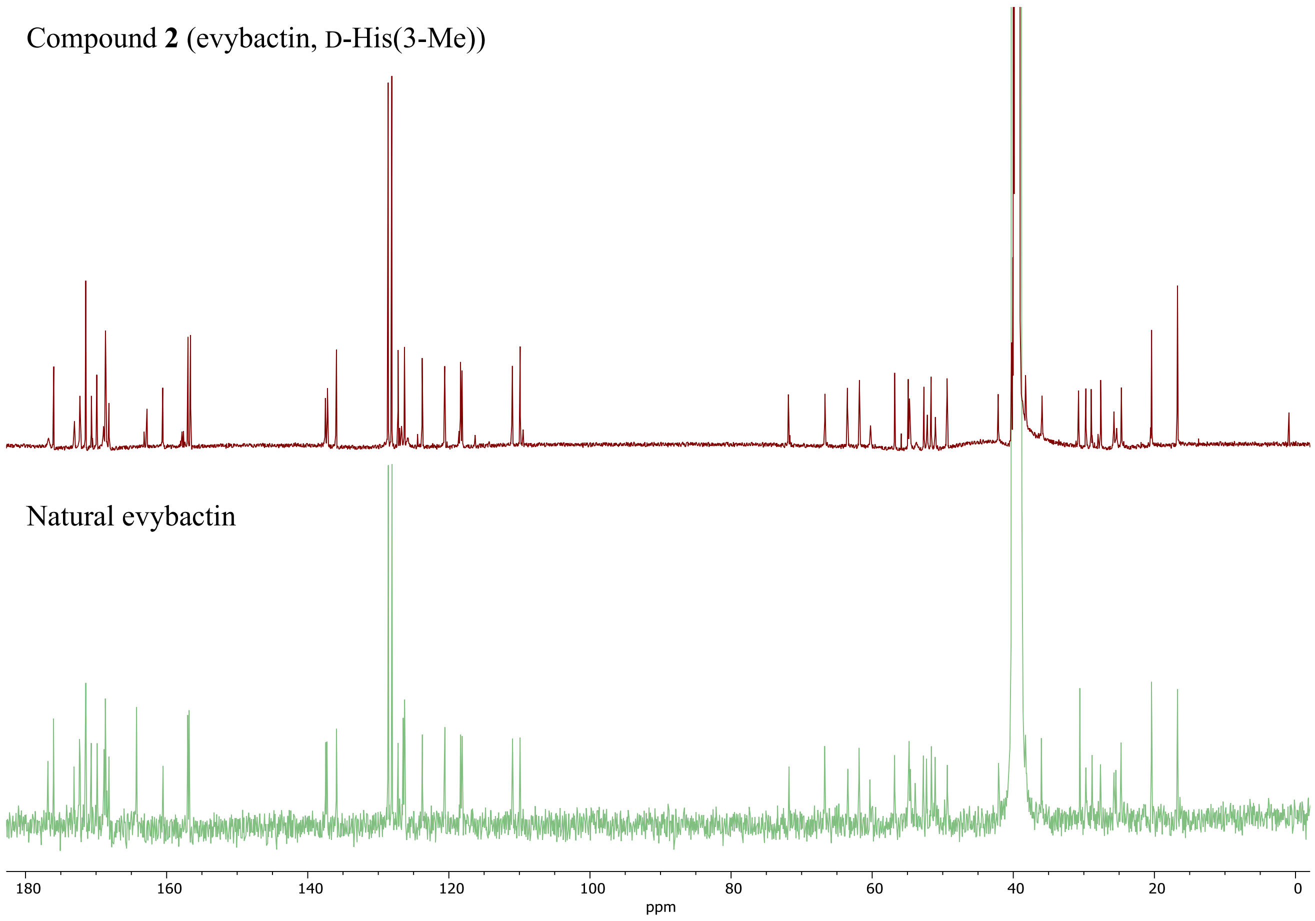

Figure S3. Full comparison (180 – 0 ppm) of the previously published ^{13}C-NMR (101 MHz, DMSO-d_6, 320K) spectrum of natural evybactin (as formic acid salt) isolated after fermentation of the producing organism overlaid with ^{1}H-NMR (151 MHz, DMSO-d_6, 320K) spectra of synthetic compound **2** (as a formic acid salt). The small difference between the NMR of compound **2** and that of natural evybactin can be explained by variations in the compound concentration and the content of water in the sample.

Analysis of the biosynthetic gene cluster

The genome sequence of *Photorhabdus noenieputensis* DSM 25462 (GenBank accession number: GCA_023108895.1) was submitted to antiSMASH 6.0 software.[26] The biosynthetic gene cluster was identified as non-ribosomal peptide synthetases harboring total of 12 modules by prediction of the adenylation domain specificity. Analysis of module 5 incorporating the Me-His residue was further analyzed by BlastP to confirm the presence of methyltransferase domain.

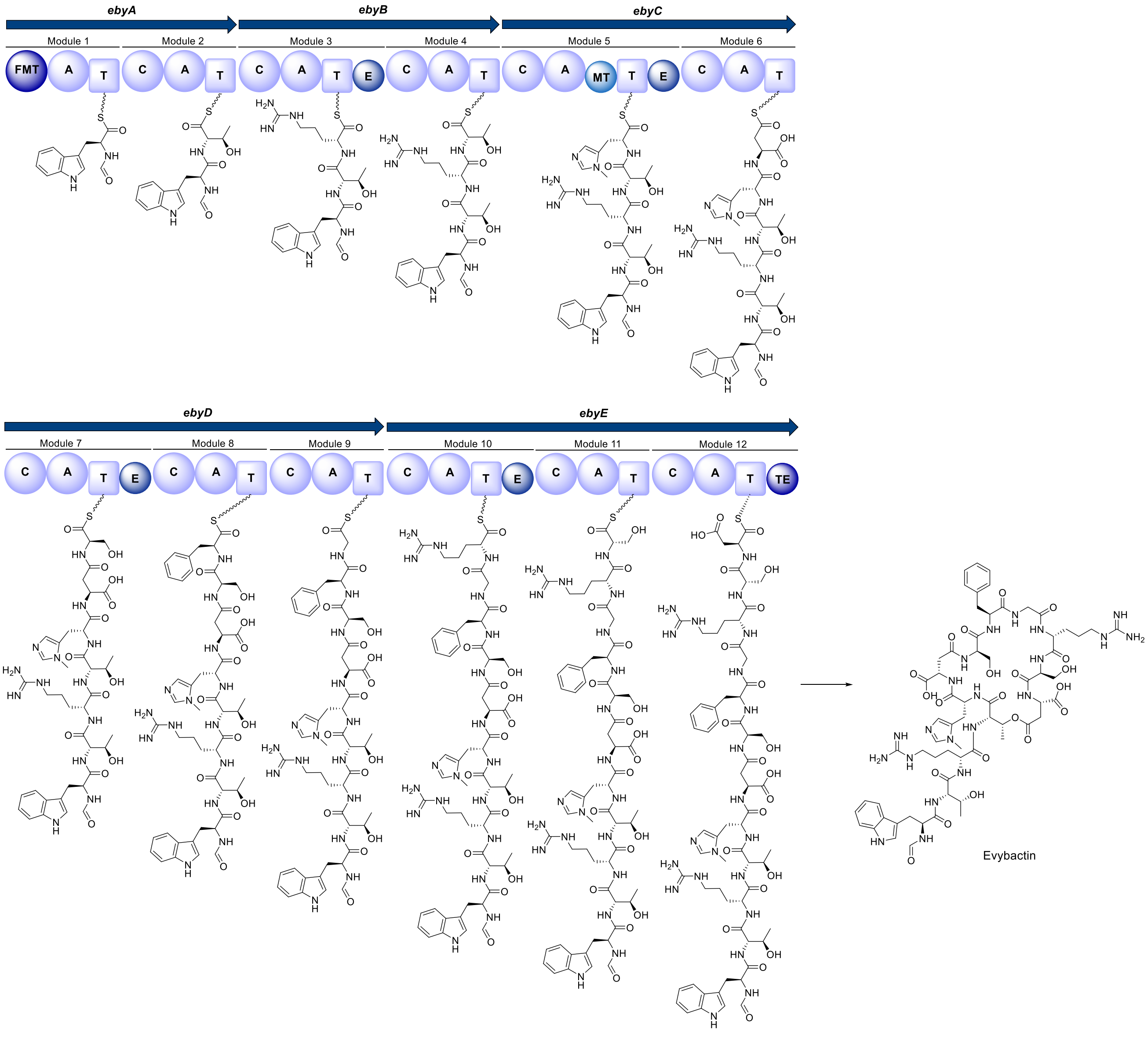

Figure S4. Schematic representation of evybactin biosynthesis (C, condensation; A, adenylation; T, thiolation; E, epimerization; MT, methyltransferase; TE, thioesterase; FMT, formyltransferase domain).

NMR Spectra

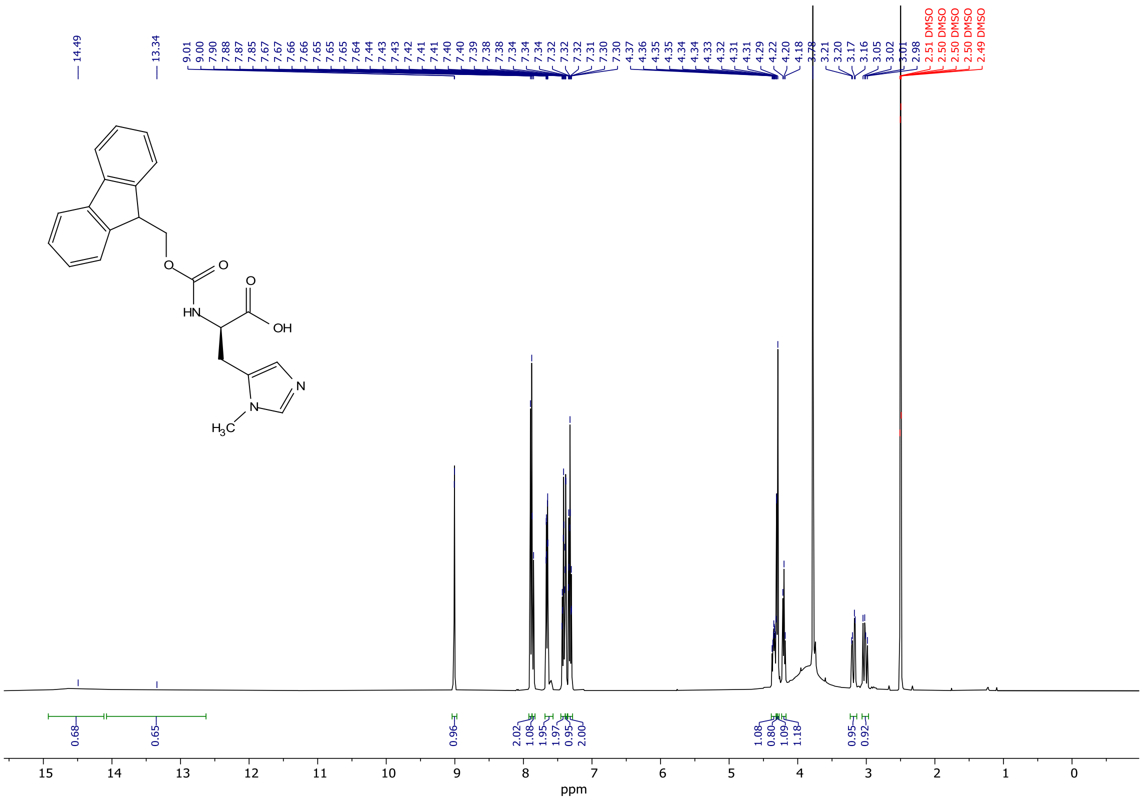

Figure S5. ^{1}H-NMR (400 MHz, DMSO-*d*$_6$, 298K) of Fmoc-D-His(3-Me)-OH (**12**)

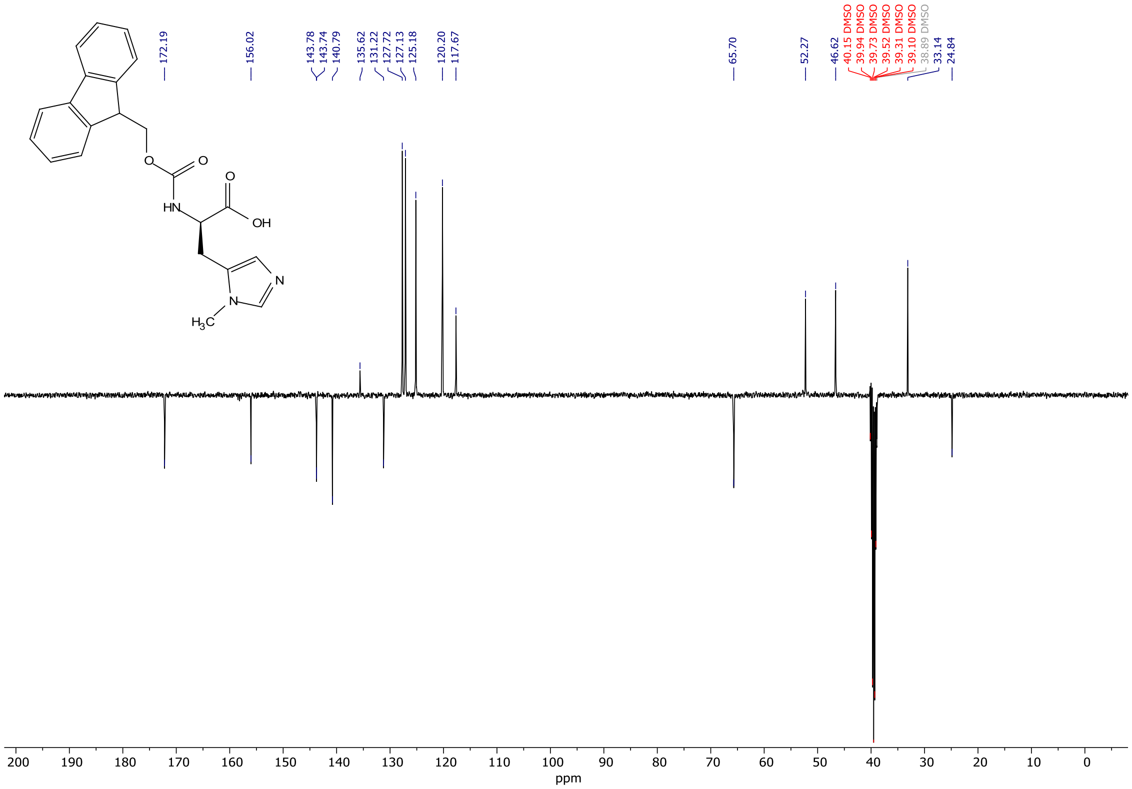

Figure S6. ^{13}C-NMR (101 MHz, DMSO-*d*$_6$, 298K) of Fmoc-D-His(3-Me)-OH (**12**)

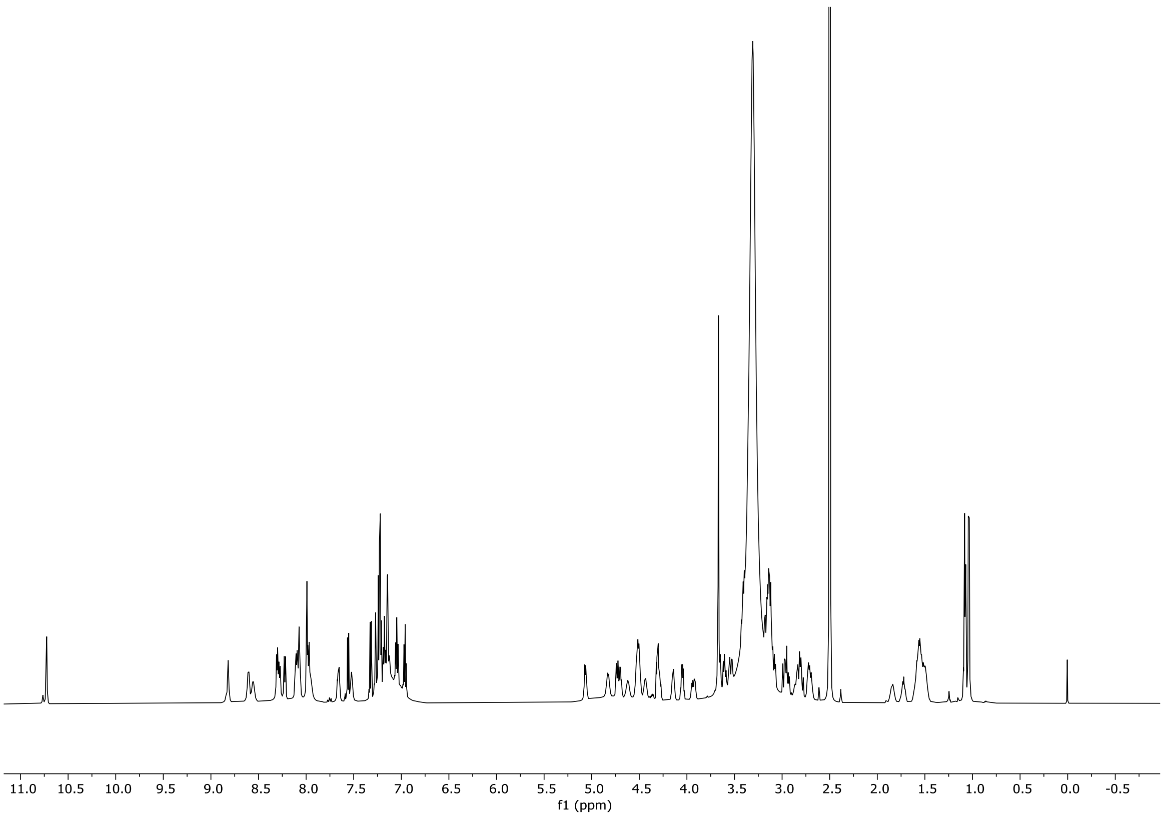

Figure S7. ^{1}H-NMR (600 MHz, DMSO-d_6, 320K) of the compound **1** as a TFA salt

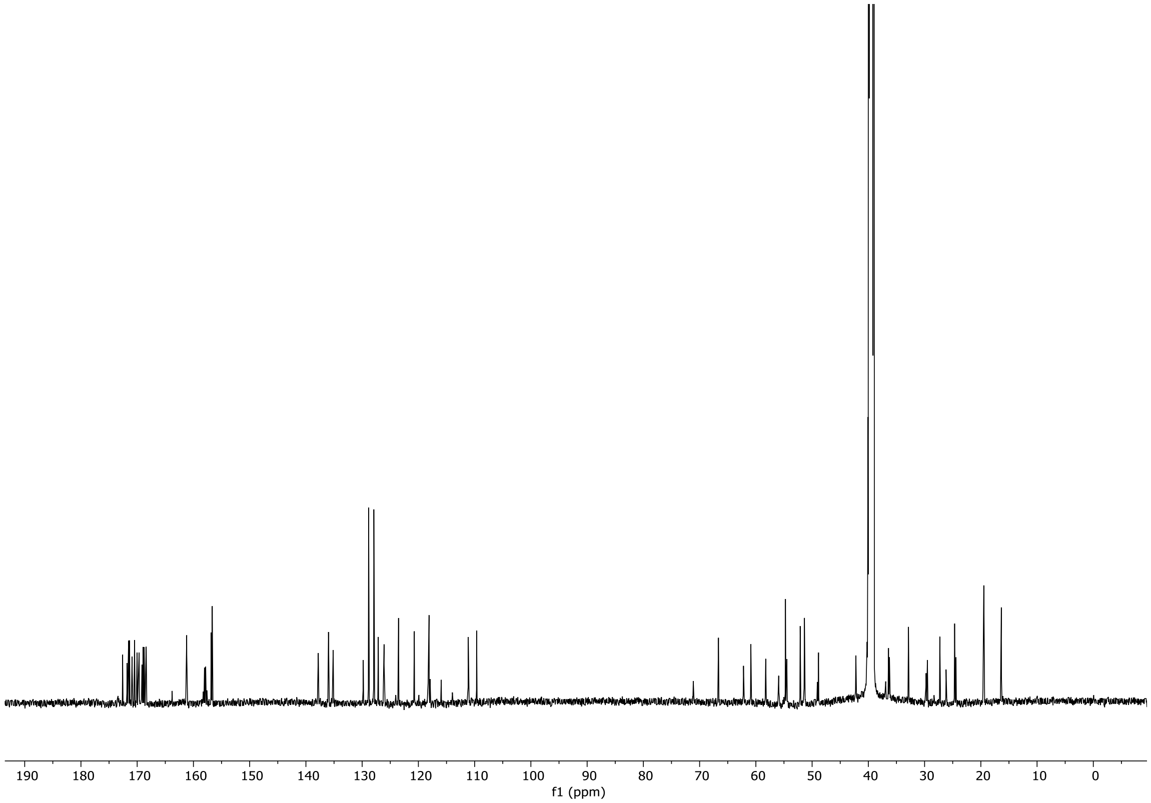

Figure S8. ^{13}C-NMR (151 MHz, DMSO-d_6, 320K) of the compound **1** as a TFA salt

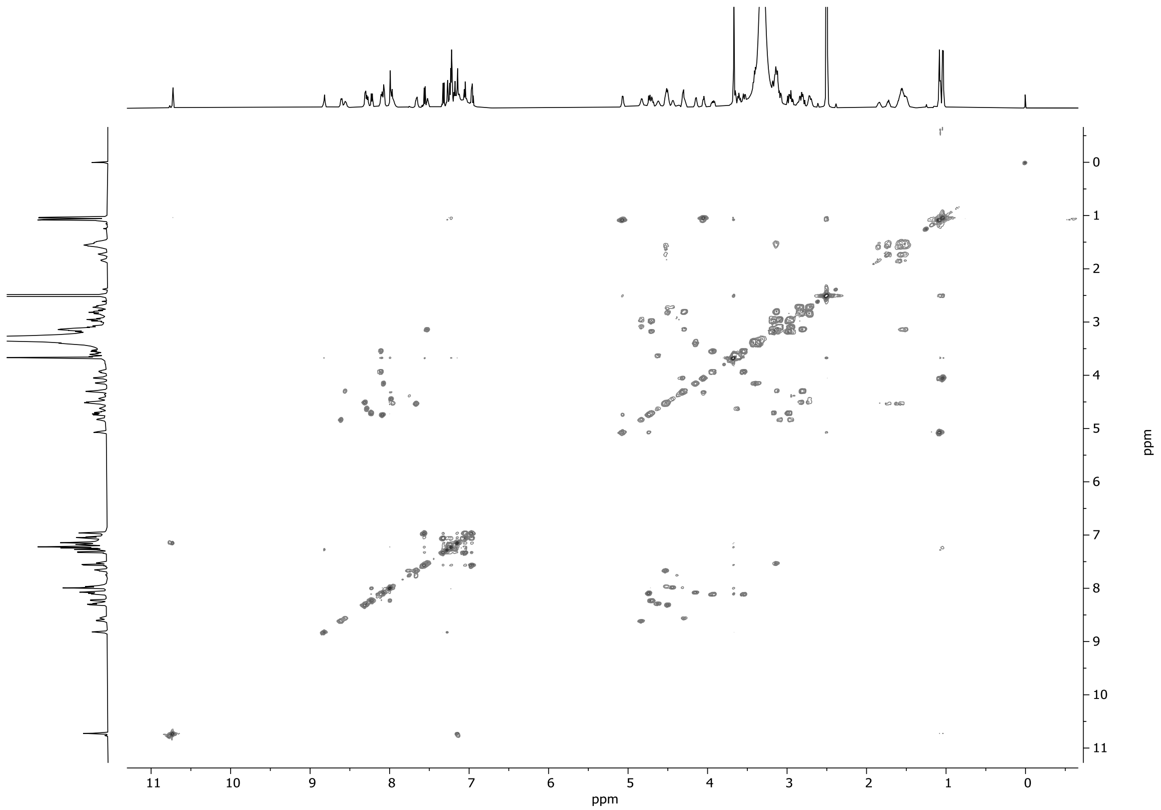

Figure S9. ^{1}H-^{1}H COSY NMR (600 MHz, DMSO-d_6, 320K) of the compound **1** as a TFA salt

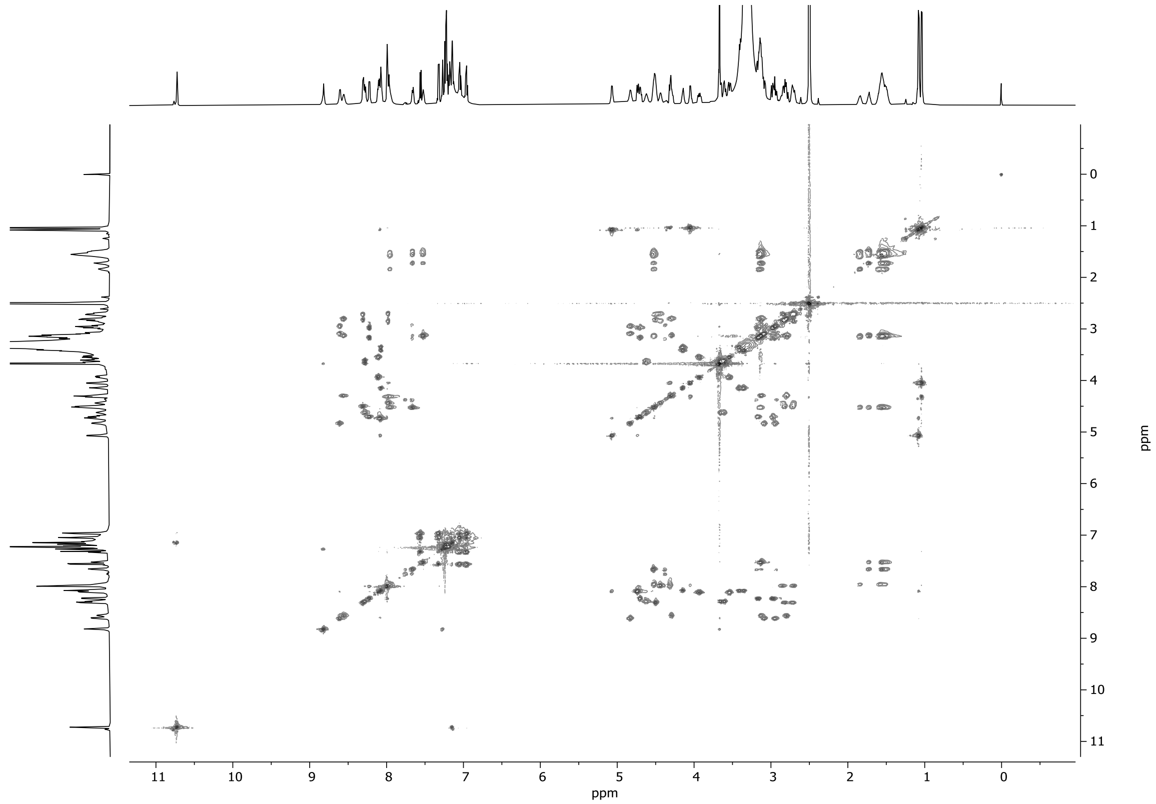

Figure S10. ^{1}H-^{1}H TOCSY NMR (600 MHz, DMSO-d_6, 320K) of the compound **1** as a TFA salt

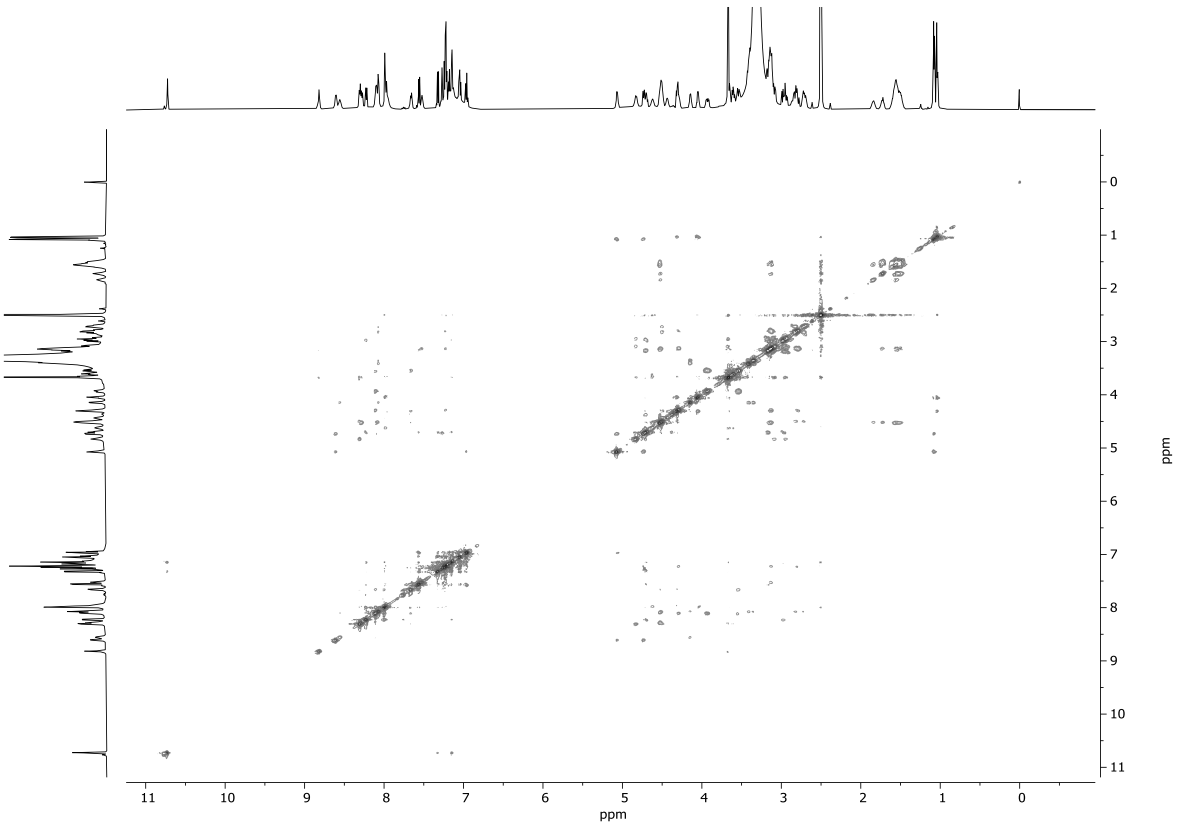

Figure S11. ^{1}H-^{1}H ROESY NMR (600 MHz, DMSO-d_6, 320K) of the compound **1** as a TFA salt

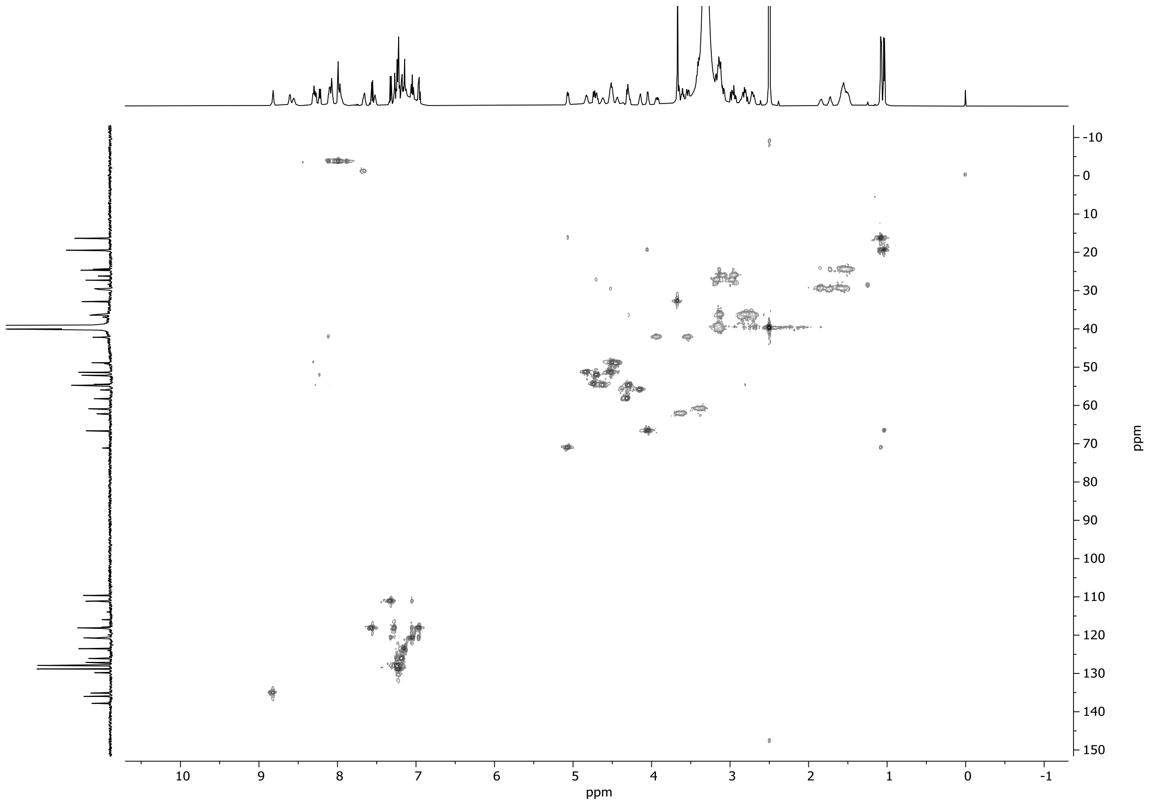

Figure S12. ^{1}H-^{13}C HSQC NMR ((600, 151) MHz, DMSO-d_6, 320K) of the compound **1** as a TFA salt

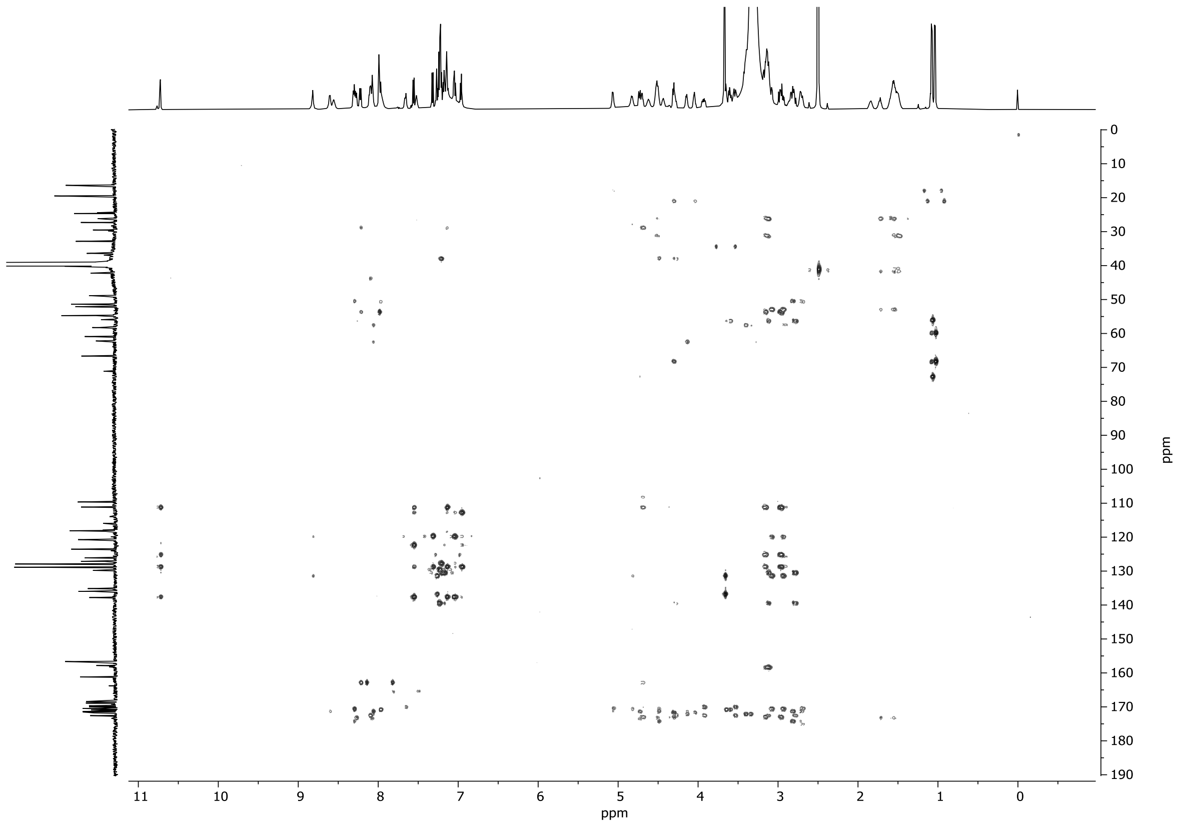

Figure S13. ^{1}H-^{13}C HMBC NMR ((600, 151) MHz, DMSO-d_6, 320K) of the compound **1** as a TFA salt

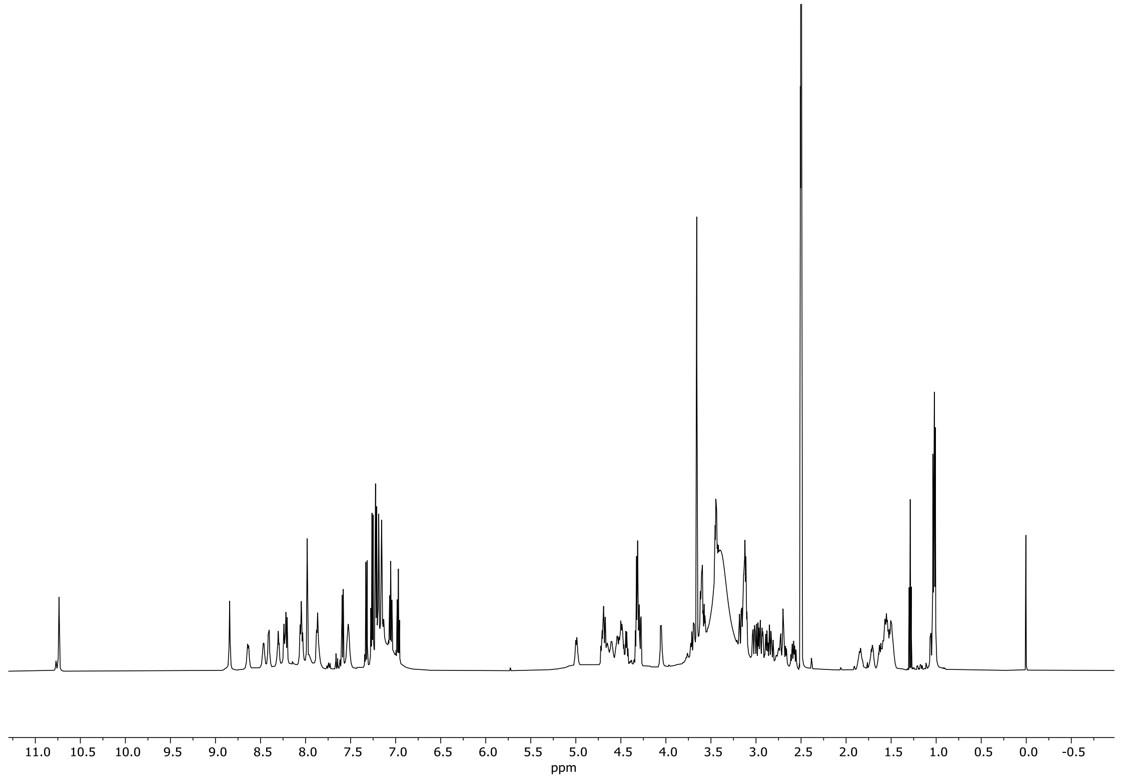

Figure S14. ^{1}H-NMR (600 MHz, DMSO-d_6, 320K) of the compound **2** as a TFA salt

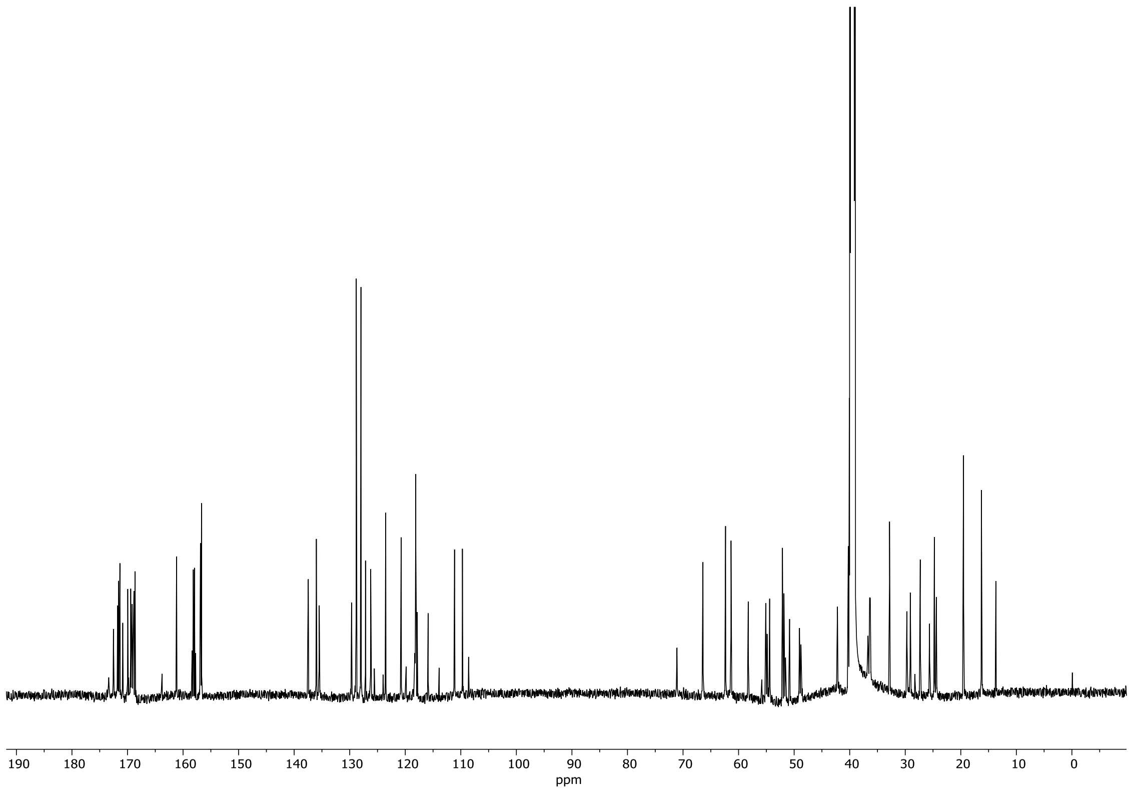

Figure S15. ^{13}C-NMR (151 MHz, DMSO-d_6, 320K) of the compound **2** as a TFA salt

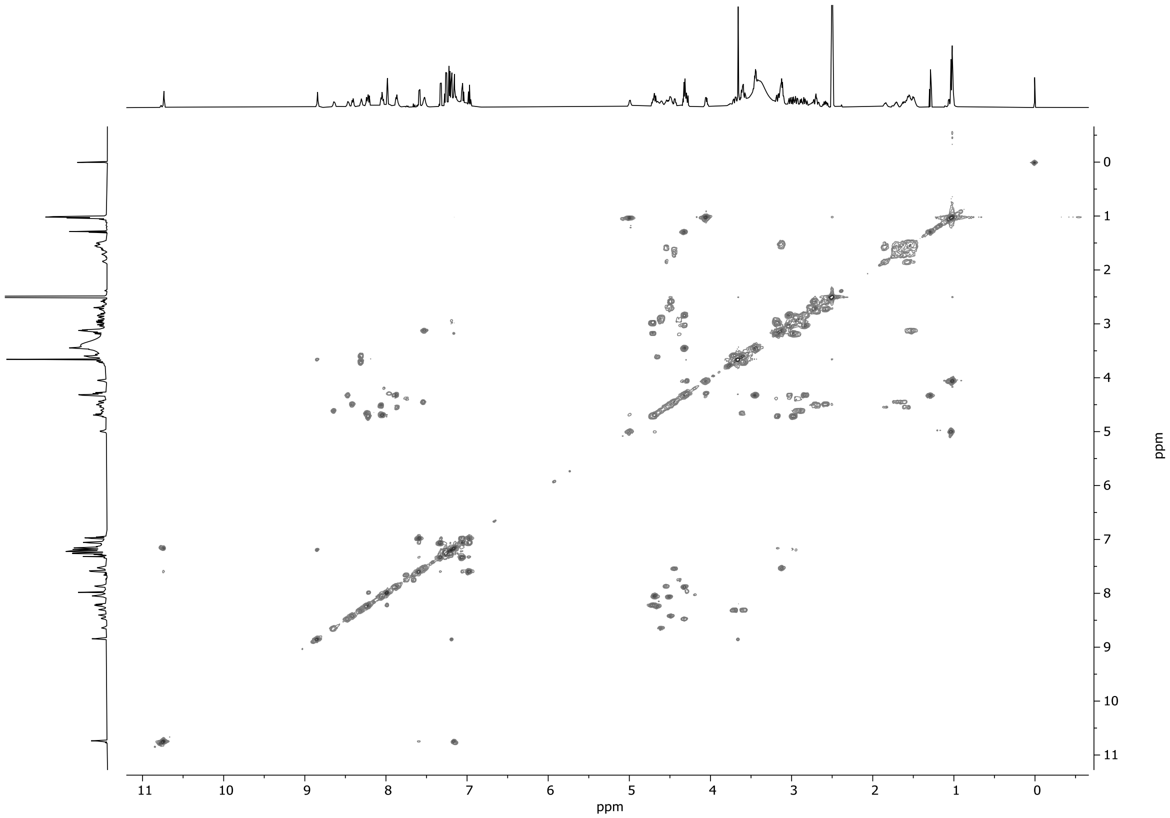

Figure S16. ^{1}H-^{1}H COSY NMR (600 MHz, DMSO-d_6, 320K) of the compound **2** as a TFA salt

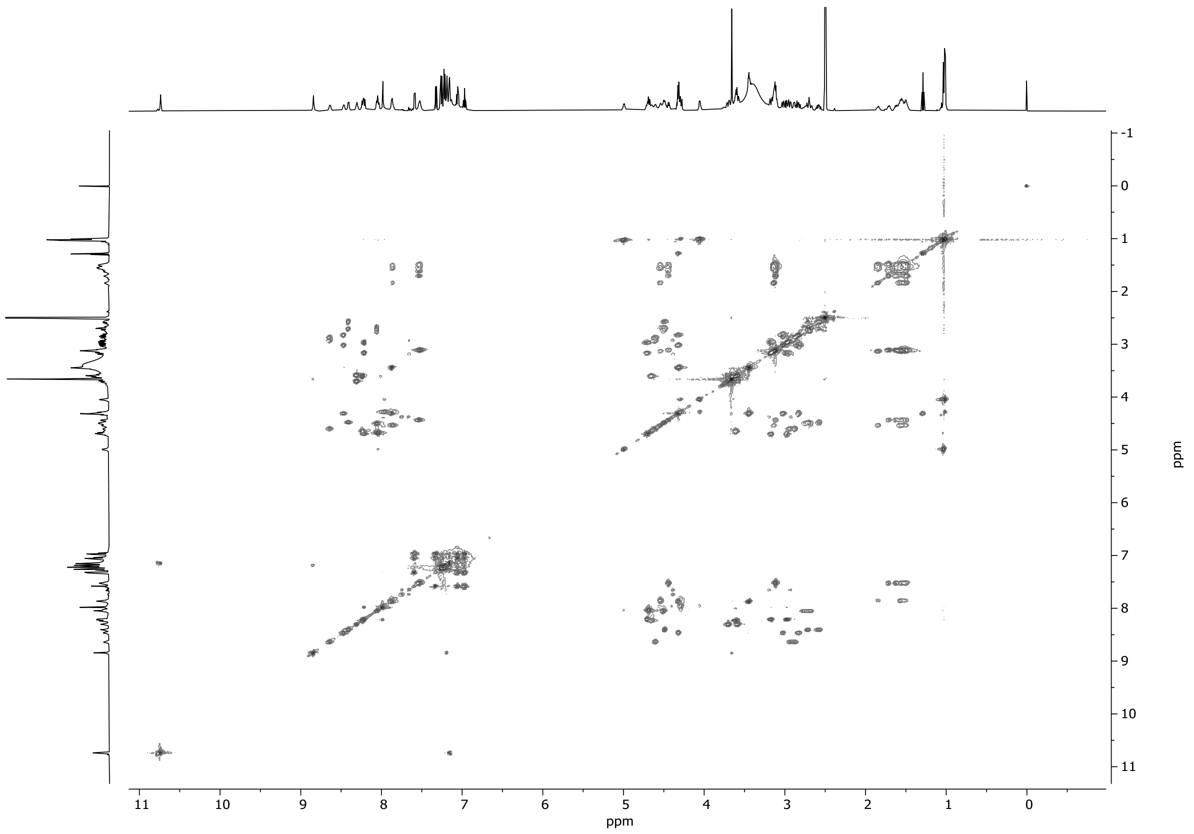

Figure S17. ^{1}H-^{1}H TOCSY NMR (600 MHz, DMSO-d_6, 320K) of the compound **2** as a TFA salt

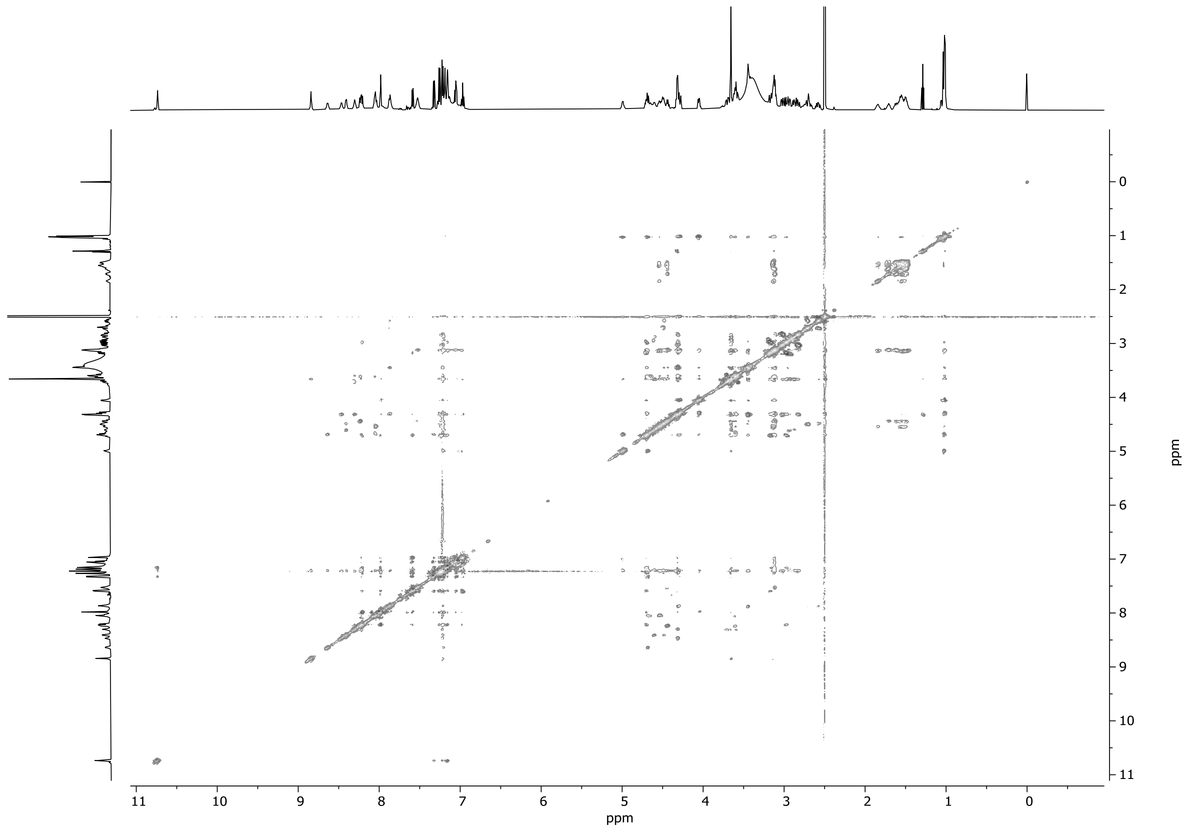

Figure S18. ^{1}H-^{1}H ROESY NMR (600 MHz, DMSO-d_6, 320K) of the compound **2** as a TFA salt

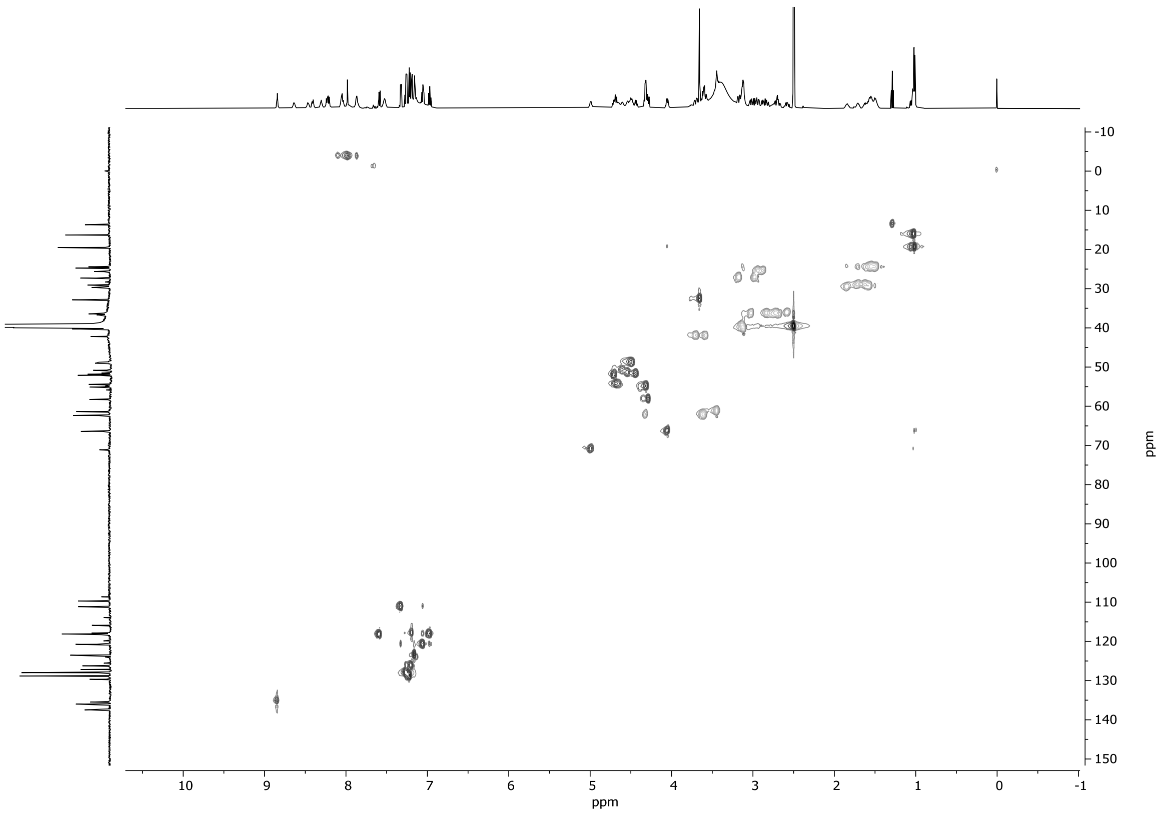

Figure S19. ^{1}H-^{13}C HSQC NMR ((600, 151) MHz, DMSO-d_6, 320K) of the compound **2** as a TFA salt

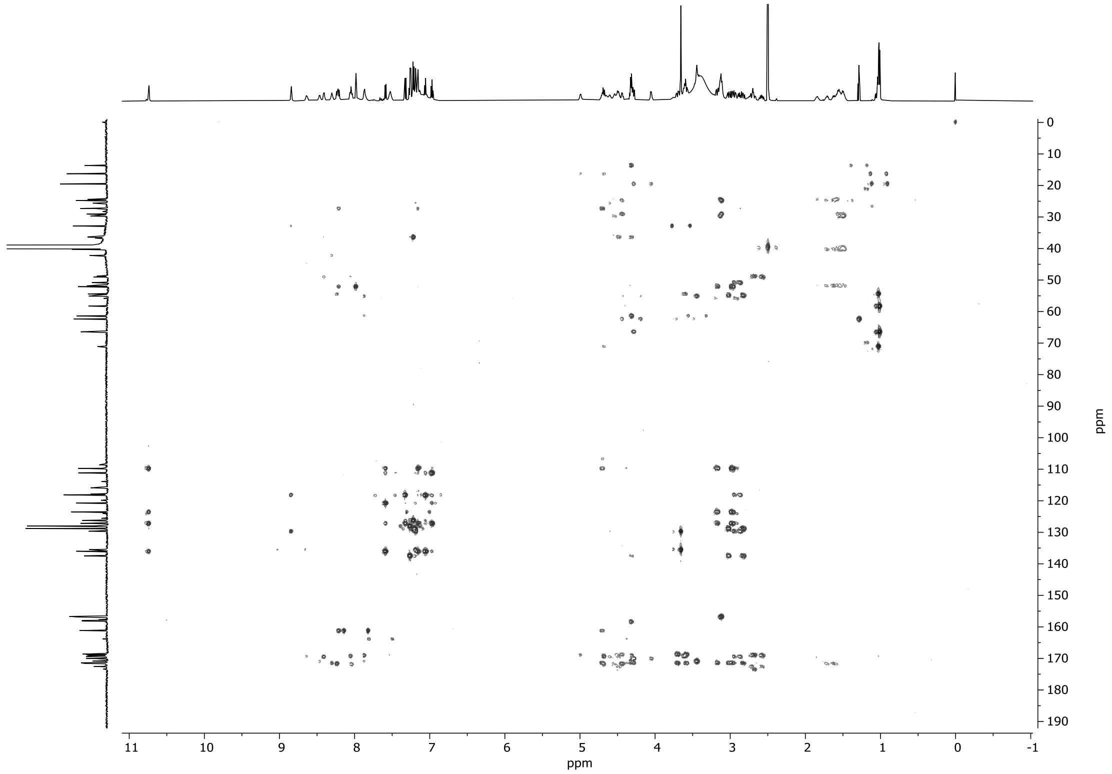

Figure S20. ^{1}H-^{13}C HMBC NMR ((600, 151) MHz, DMSO-d_6, 320K) of the compound **2** as a TFA salt

HPLC analysis of peptides

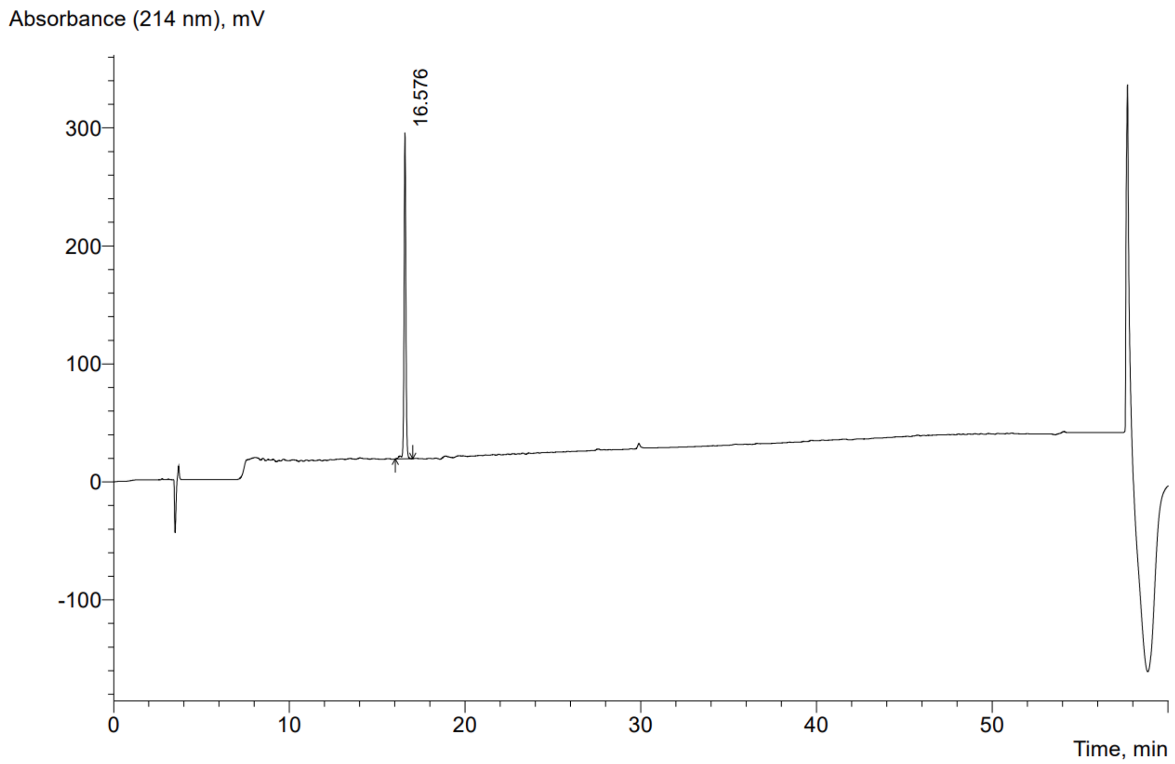

Figure S21. HPLC trace of the purified compound **1**. The peptide eluted as a single peak at 16.58 min.

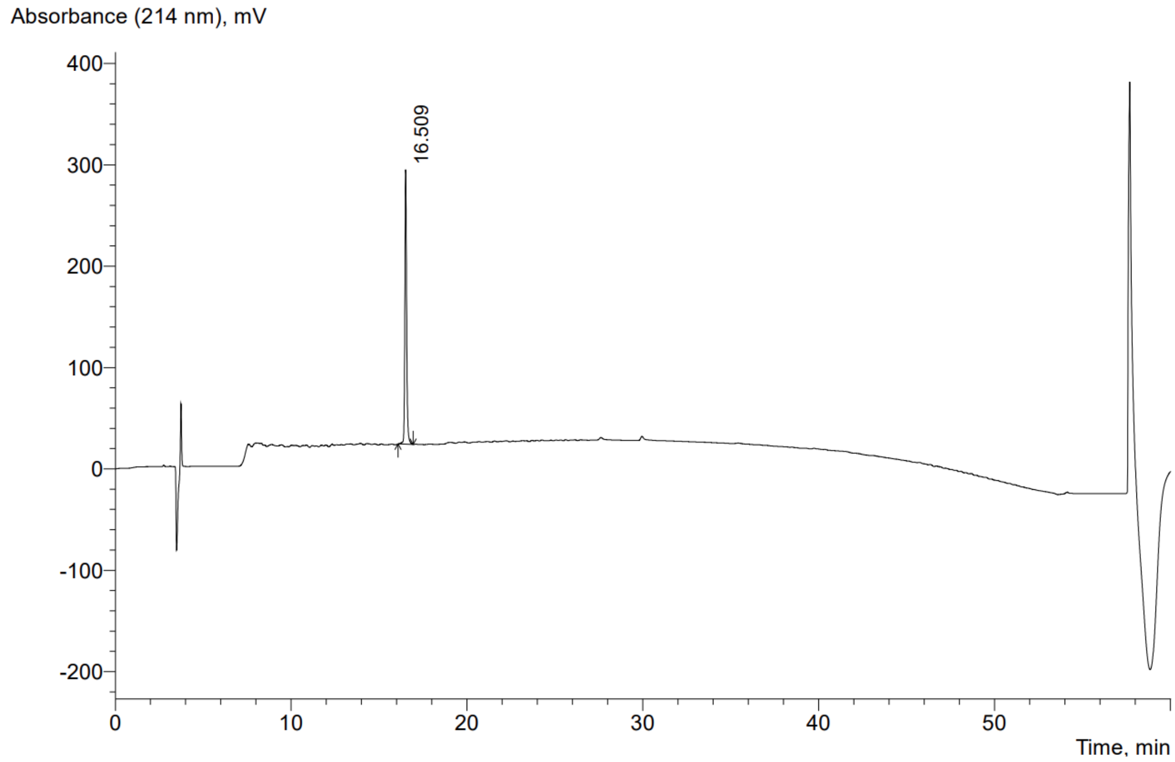

Figure S22. HPLC trace of the purified compound **2**. The peptide eluted as a single peak at 16.51 min.

References:

[9] Y. Imai, G. Hauk, J. Quigley, L. Liang, S. Son, M. Ghiglieri, M. F. Gates, M. Morrissette, N. Shahsavari, S. Niles, D. Baldisseri, C. Honrao, X. Ma, J. J. Guo, J. M. Berger, K. Lewis, *Nat Chem Biol* **2022**, *18*, 1236–1244.

[19] P. Marfey, *Carlsberg Res Commun* **1984**, *49*, 591–596.

[23] D. A. van Bergeijk, S. S. Elsayed, C. Du, I. N. Santiago, A. M. Roseboom, L. Zhang, V. J. Carrión, H. P. Spaink, G. P. van Wezel, *Commun Chem* **2022**, *5*, 14.

[24] R. Schmid, S. Heuckeroth, A. Korf, A. Smirnov, O. Myers, T. S. Dyrlund, R. Bushuiev, K. J. Murray, N. Hoffmann, M. Lu, A. Sarvepalli, Z. Zhang, M. Fleischauer, K. Dührkop, M. Wesner, S. J. Hoogstra, E. Rudt, O. Mokshyna, C. Brungs, K. Ponomarov, L. Mutabdžija, T. Damiani, C. J. Pudney, M. Earll, P. O. Helmer, T. R. Fallon, T. Schulze, A. Rivas-Ubach, A. Bilbao, H. Richter, L. F. Nothias, M. Wang, M. Orešič, J. K. Weng, S. Böcker, A. Jeibmann, H. Hayen, U. Karst, P. C. Dorrestein, D. Petras, X. Du, T. Pluskal, *Nat Biotechnol* **2023**, *41*, 447–449.

[25] S. Vijayasarathy, P. Prasad, L. J. Fremlin, R. Ratnayake, A. A. Salim, Z. Khalil, R. J. Capon, *J Nat Prod* **2016**, *79*, 421–427.

[26] K. Blin, S. Shaw, A. M. Kloosterman, Z. Charlop-Powers, G. P. Van Wezel, M. H. Medema, T. Weber, *Nucleic Acids Res* **2021**, *49*, W29–W35.
